# Wearable Bioelectronics for Home‐Based Monitoring and Treatment of Muscle Atrophy

**DOI:** 10.1002/advs.202502831

**Published:** 2025-07-12

**Authors:** Shuai Zhang, Renjie Tan, Shuo Meng, Ke Zhang, Jinlian Hu

**Affiliations:** ^1^ Department of Biomedical Engineering City University of Hong Kong Kowloon Hong Kong SAR 999077 China

**Keywords:** bioelectronics, electrode–tissue interfaces, monitoring, muscle atrophy, treatment

## Abstract

Muscle atrophy is a chronic disease caused by multiple factors, and there is currently no effective treatment or reliable diagnostic method. Similar review articles focus on the induction and summary of the pathogenic mechanism and traditional treatment of muscle atrophy. However, soft, portable, and efficient bioelectronic devices that are expected to achieve home diagnosis and treatment of the disease are more concerned, hoping to realize complete home care as soon as possible. The article includes: 1) Classification and summary of muscle atrophy‐related diseases and pathways; 2) Mechanical sensors, electromyography (EMG), electrochemical sensors, bioelectrical impedance analysis (BIA), and ultrasonic patches for monitoring muscle atrophy; 3) Transcutaneous electrotherapy, bioelectronic implants, bioelectronic drug delivery devices, and ExoMuscles for treating muscle atrophy; 4) Electrode–tissue interface problems in muscle atrophy bioelectronics and feasible solutions. 5) Recommendations and prospects for integrated bioelectronic systems for home diagnosis and treatment of muscle atrophy in the future.

## Graphical abstract

As an inevitable disease, muscle atrophy has received more attention. Because the factors that induce this disease are diverse, achieving a complete cure is still impossible. Wearable bioelectronics provides a more comfortable, low‐cost, and efficient way of home care for the monitoring and treatment of muscle atrophy. Therefore, this review summarizes and looks forward to the development of this field.

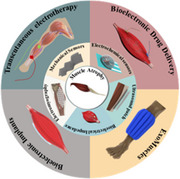

## 1 Introduction

Muscle atrophy involves the loss of muscle mass and strength. There are many known causes, including genetic defects, aging (sarcopenia), chronic diseases (such as cancer, chronic obstructive pulmonary disease, and heart failure), poor nutrition, lack of exercise, and specific medical treatments (prolonged bed rest or use of corticosteroids).^[^ ^1^, ^2^, ^3^ ^]^ Muscle atrophy can significantly impact an individual's quality of life, leading to decreased mobility, increased risk of falls and fractures, and overall decline in physical function. Sarcopenia affects about 10% of adults over 60 and 50% of those over 80 in the aging population, with higher rates among those with chronic illness or hospitalization. In addition, there are more than a dozen genetic diseases (such as spinal muscle atrophy (SMA), Duchenne muscular dystrophy (DMD), and amyotrophic lateral sclerosis (ALS)) that cause muscle atrophy, most of which are fatal.^[^ ^4^, ^5^, ^6^, ^7^, ^8^ ^]^ Moreover, traumatic peripheral nerve injuries affect more than 350 000 patients each year in the United States alone.^[^ ^9^ ^]^ In short, muscle atrophy has a wide range of impacts on people's lives and health.

Therefore, this disease has become a heavy burden on medical care in countries or regions worldwide. A study by Sophelia H. S. Chan^[^ ^10^ ^]^ showed that SMA alone cost Hong Kong patients an average of US$935570 in cumulative medical expenses, with type 1 patients spending as much as US$2393250 up to 18. Targeted therapies for DMD have cost the U.S. healthcare system more than $3 billion.^[^ ^11^ ^]^ The musculoskeletal system is among the most burdensome diseases in the Czech Republic. The average annual healthcare costs associated with muscle weakness in older adults without long‐term diseases were estimated to be €1125.3 ± 1367.2.^[^ ^12^ ^]^

**Figure 1:**
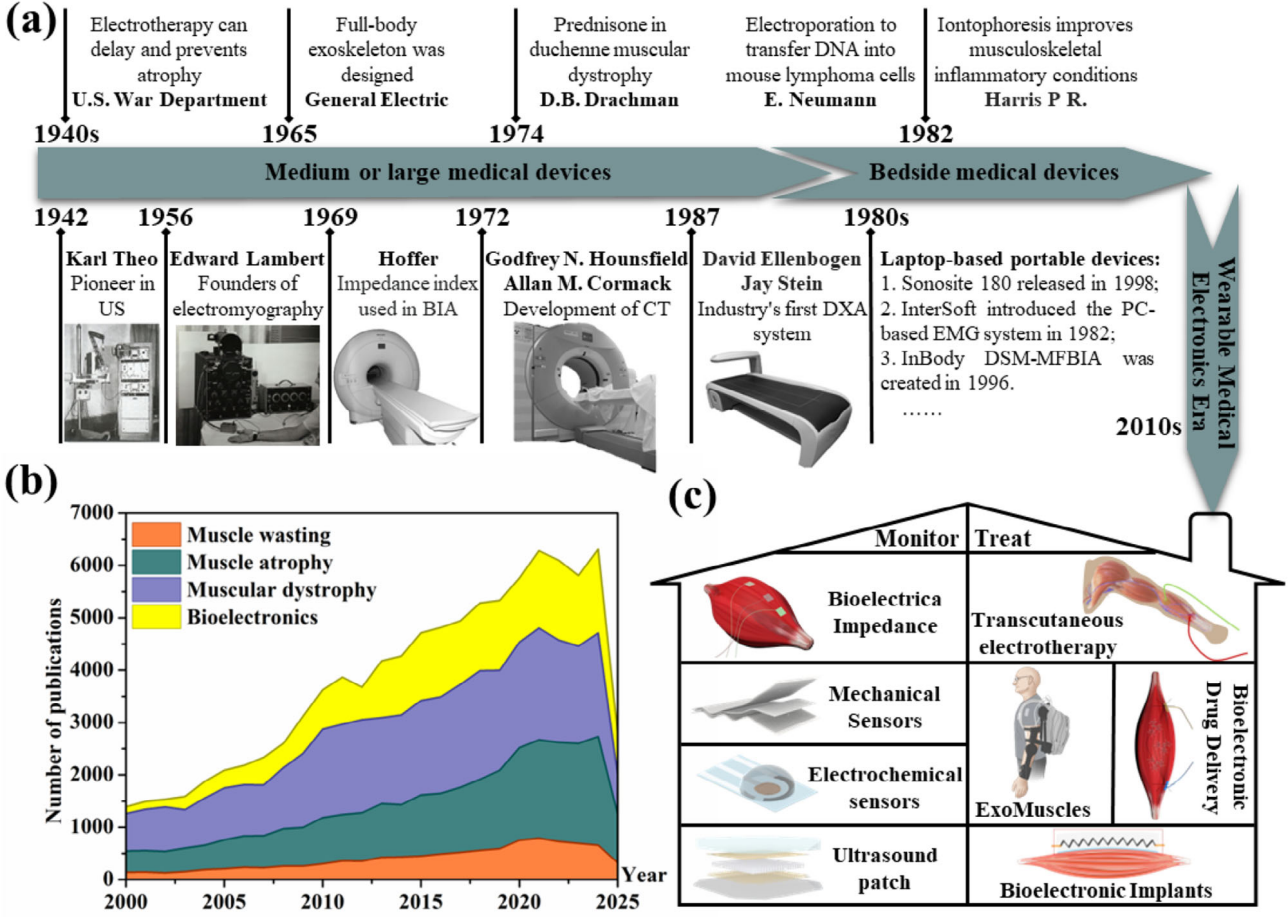
a) A chronological diagram of the emergence of methods or instruments for monitoring and treating muscle atrophy. Reproduced with permission.^[^ ^17^ ^]^ Copyright 2024, Wiley. b) The number of relevant publications retrieved on the Web of Science changes over time (Until June 2025). c) Summary of technical methods for home monitoring and treatment of muscle atrophy. Reproduced with permission.^[^ ^290^ ^]^ Copyright 2025, ACS. Reproduced with permission.^[^ ^156^ ^]^ Copyright 2022, Science. Reproduced with permission.^[^ ^229^ ^]^ Copyright 2022, Springer Nature. Reproduced with permission.^[^ ^268^ ^]^ Copyright 2024, Springer Nature.

Muscle atrophy poses a serious health threat and heavy economic pressure to patients. Related cases have been recorded as early as 1830, but no unified and effective diagnosis and treatment plan is currently available. Clinical detection of the disease mainly relies on imaging techniques^[^ ^13^, ^14^, ^15^, ^16^, ^17^, ^18^ ^]^ such as MRI (magnetic resonance imaging), CT (computed tomography), DXA (dual‐energy X‐ray absorptiometry), US (ultrasound), and some auxiliary strength tests. Although the laboratory has discovered many targets for muscle atrophy with different causes, there is currently no effective treatment for the disease.^[^ ^2^, ^19^ ^]^ It has been found that the means that can effectively prevent and assist muscle atrophy include electrical stimulation (ES), exoskeletons, drugs, and electrically assisted drug release.^[^ ^20^, ^21^, ^22^, ^23^ ^]^ We list the key dates for diagnosing and treating atrophy since the 1940s in **Figure** **1**a and divide them into three stages: medium or large medical devices, bedside medical devices, and the wearable medical electronics era.

Wearable bioelectronics plays a vital role in home diagnosis and treatment, covering areas such as tissue repair,^[^ ^24^, ^25^, ^26^, ^27^ ^]^ disease treatment,^[^ ^28^, ^29^, ^30^ ^]^ health monitoring,^[^ ^31^, ^32^, ^33^, ^34^, ^35^ ^]^ and human–computer interaction.^[^ ^36^, ^37^, ^38^ ^]^ In addition, the statistics of publication retrieval in muscle atrophy (Figure 1b) also show that the research on this disease is gaining more and more attention. However, given that there is no cure at this stage and the heavy medical pressure, home care has become the best solution. The key difference between this review and similar articles is the summary and prospect of wearable technologies that have the potential to achieve home diagnosis and treatment of muscle atrophy. Monitoring technologies are divided into five types: mechanical sensors, EMG, electrochemical sensors, US patches, and BIA, as shown in Figure 1c. Treatment methods are divided into four categories: transcutaneous electrotherapy, bioelectronic implants, bioelectronic drug delivery, and ExoMuscles, as shown in Figure 1c. Unlike summaries of pathogenic mechanisms^[^ ^39^, ^40^ ^]^ and traditional treatments,^[^ ^23^, ^41^ ^]^ our review focuses on the latest research trends and technological innovations expected to achieve home diagnosis and treatment. It also emphasizes the potential for future development and will become a valuable professional resource.

## 2 Physiological Mechanism of Muscle Atrophy

Muscles usually account for about 30% to 40% of an adult's body weight and are one of the most critical tissues in the human body. They can be divided into skeletal muscle, cardiac muscle, and smooth muscle according to their structure, physiological function, and type of nerve innervation. Among them, skeletal muscle, which is used to maintain posture, perform movements, and protect internal organs, is the focus of our explanation. Nerves control skeletal muscle movements, so their damage may lead to diseases, including muscle atrophy.^[^ ^42^, ^43^, ^44^ ^]^ Nerves and skeletal muscles originate from the ectoderm and mesoderm of the embryo, respectively. When genetic genes are damaged, it will induce muscle atrophy, which is mostly fatal. In addition, nerve damage, inflammation, disuse, CKD, etc., may also cause muscle atrophy. More diseases associated with muscle atrophy and their characteristics, including detection and treatment methods, are summarized in **Table** **1**.

**Table 1:** A summary of the symptoms, causes, incidence, testing methods, and treatment options for muscle atrophy disorders.

Category	Disease	Symptom	Pathogen	Incidence	Diagnosis method	Treatment	Ref.
Congenital muscle atrophy	SMA	Severe muscle weakness and often early death	SMN1 mutations	1/5000 to 1/10 000 live births in Europe	–	Gene therapy, cell therapy, neuroprotection, muscle enhancement	[5, 49]
	DMD	Muscle wasting	DMD mutation	Less than 1/10 000 males	Plasma creatine kinase, multiplex PCR, muscle biopsy	Gene therapy, cell therapy, muscle protection	[4]
	ALS	Muscle weakness, weight loss, and muscle loss	C9orf72 is the most commonly known	1.68 per 100 000 person‐years	Neuro exam, electrodiagnostic testing, MRI	Riluzole, edaravone, genetic therapies, antibodies	[6]
	Charcot‐Marie‐Tooth (CMT)	Distal muscle weakness and atrophy, sensory loss, hyporeflexia, and skeletal deformity	More than 40 genes expressed in Schwann cells and neurons are mutated	17 to 40/10 000	Genetic testing, genetic counseling	Genetic therapies	[7]
	Giant axonal neuropathy (GAN)	Hypotonia, muscle atrophy, tendon contractures, and areflexia along with signs of central nervous system involvement	Biallelic loss‐of‐function variants in GAN	–	Muscle biopsies, MRI, muscle biopsies	Gene therapy, symptomatic treatment	[50, 51, 52]
	Congenital myasthenic syndrome (CMS)	Respiratory involvement, bulbar weakness, hypotonia, with or without muscle weakness	35 genes reported to be associated with CMS	2.8–14.8 per million (UK), 1.8 per million (Brazil), and 22.2 per million (Slovenia)	EMG, DNA sequencing	Drug, gene replacement therapies	[8]
	Bethlem myopathy	Congenital weakness and hypotonia	Mutations in the collagen VI genes	0.77 per 100 000	MRI, muscle biopsies, muscle ultrasonography, molecular genetic analysis, genetic counseling	Drug	[53]
Acquired muscle atrophy	Myasthenia Gravis (MG)	Muscle fatigue with a degree of weakness	Antibody‐mediated autoimmune disorders	5.3 per million person‐years	Serologic or electrodiagnostic testing, cell‐based assays, MRI	Surgical therapy, pharmacological therapy, stem cell therapies, Interference with cell signaling	[54, 55]
	Idiopathic inflammatory myopathies (IIM)	Muscle weakness, muscle shrinking, and loss of muscle	Immune‐mediated muscle injury	–	MRI, muscle biopsies, myositis damage index, antibody testing	Drugs, physical exercise	[56, 57]
	Guillain–Barre syndrome (GBS)	Infectious illness, limb weakness	Immune‐mediated polyradiculoneuropathy	Estimated 100 000 new cases annually worldwide	Nerve conduction studies, cerebrospinal fluid analysis, MRI, nerve US	Immunotherapy, intravenous immunoglobulin, plasma exchange, physiotherapy	[58]
	Chronic kidney disease (CKD)	Anemia, hyperkalemia, Decline in muscle strength and function, etc.	Chronic catabolic disease	Global prevalence is 9.1%^[^ ^59^ ^]^	glomerular filtration rate, albumin‐creatinine ratio	Drugs, exercise, renal replacement therapies	[41]
	Aging	Decline in muscle mass and function	Mitochondrial and autophagy dysfunction, lack of satellite cell regeneration ability, etc.	–	DXA	Physical exercise, nutrition, and supplements	[60]
	Muscle disuse	Loss of muscle mass and strength	Altered mitochondrial dynamics, changes of morphology and function, and redox imbalance, etc.	–	EMG, serum detection, biopsy	Tea polyphenols, physical exercise, ES, nutrition, drugs	[44, 61, 62]
	Parkinson's disease	Frailty and decreased muscle mass	Loss of nigrostriatal dopaminergic neurons	Affect ≈1% of the population over 60 years of age	MRI, genetic testing	Drugs, high‐frequency stimulation	[63, 64]

**Figure 2:**
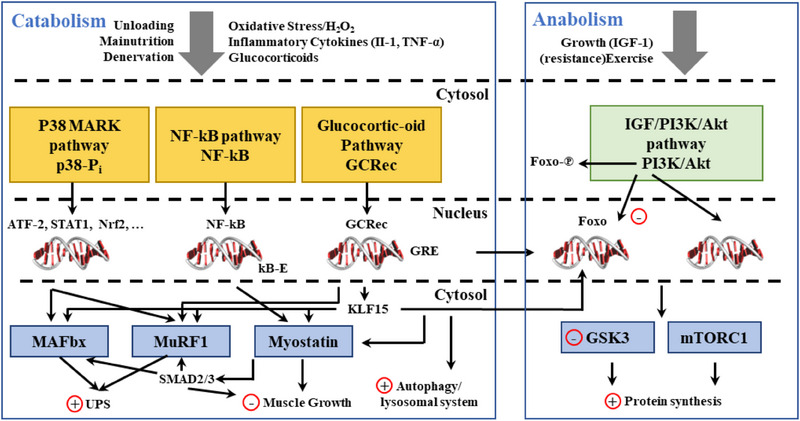
Some of the signaling pathways that affect muscle protein synthesis and breakdown. Reproduced with permission.^[^ ^48^ ^]^ Copyright 2021, Elsevier.

Exploring the various targets associated with the disease is necessary to treat muscle atrophy, which has many causes. The following are the key genes and signaling pathways related to muscle atrophy that have been discovered. The MSTN gene encodes myostatin, a major negative regulator of muscle growth, and overexpression can lead to muscle atrophy.^[^ ^45^ ^]^ UBR2 (Ubiquitin Protein Ligase E3 Component N‐Recognin 2) is a gene related to protein degradation. Recent studies have shown that inhibiting its expression may help slow the progression of muscle atrophy.^[^ ^46^ ^]^ Increased expression levels of MuRF1 and MAFbx promote protein degradation in muscle cells, further aggravating the process of muscle atrophy.^[^ ^47^ ^]^ Furthermore, genes such as FOXO3, TNF‐α, IL‐6, P53, and SIRT1, or their expression, play an essential role in muscle atrophy, as shown in **Figure** **2**.

## 3 Monitoring

Muscle atrophy can lead to the loss of muscle mass and strength, and changes in behavior. In addition, it will cause many biochemical changes. Therefore, there are many ways and means to monitor it using various sensors. We will summarize and conclude based on the working principle, measurement indicators, sensitivity, and importance of the sensor to inspire subsequent work.

### 3.1 Mechanical Sensors

**Figure 3:**
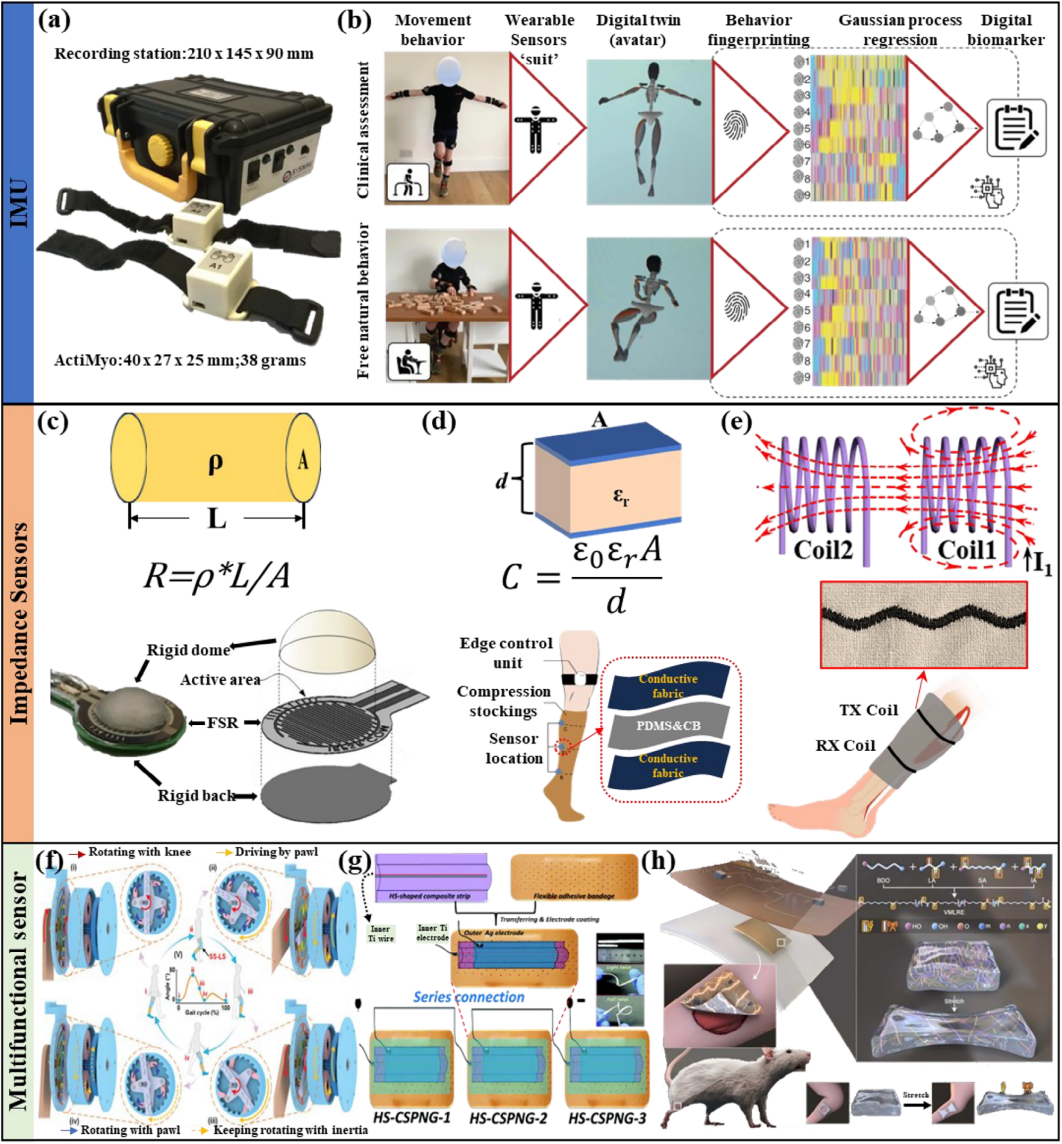
a) Optical image of ActiMyo used to monitor movement in patients with DMD. Reproduced with permission.^[^ ^67^ ^]^ Copyright 2016, PLOS. b) Overview of wearable whole‐body motion activities of daily living to extract digital biomarkers for DMD. Reproduced with permission.^[^ ^68^ ^]^ Copyright 2023, Springer Nature. c) Exploded and assembled diagram of the mechanical components of a force‐sensitive resistor‐based muscle sensor. Reproduced with permission.^[^ ^71^ ^]^ Copyright 2018, MDPI. d) Fabric sensor structure and location diagram for monitoring lower limb skeletal muscle strength. Reproduced with permission.^[^ ^72^ ^]^ Copyright 2024, MDPI. e) The principle of Faraday electromagnetic induction and textile devices for muscle atrophy detection. f) Half‐wave electromagnetic for motion joint angle detection. Reproduced with permission.^[^ ^77^ ^]^ Copyright 2023, Wiley. g) Structure and series diagram of a highly adaptable hemispherical flexible self‐powered muscle monitoring sensor. Reproduced with permission.^[^ ^78^ ^]^ Copyright 2018, Royal Society of Chemistry. h) Stretchable piezoelectric elastomers for restoration and functional monitoring of volumetric muscle loss. Reproduced with permission.^[^ ^79^ ^]^ Copyright 2023, Elsevier.

Muscle atrophy will decrease muscle strength, which in turn causes changes in the patient's behavior pattern, especially when caused by systemic progressive causes. Inertial measurement units (IMU) are widely used to detect displacement, angle, and acceleration that reflect an individual's motor ability and muscle function.^[^ ^65^, ^66^ ^]^ Data from these sensors can be used to analyze changes in movement patterns (e.g., leg muscle strength and endurance) to obtain indicators of muscle atrophy. Le Moing AG et al.^[^ ^67^ ^]^ used magnetic inertial sensors (**Figure** **3**a) to analyze that the average rotation rate and average hand‐raising rate were highly correlated with the movement state of DMD patients. However, the above experiments were conducted under conditions where the patients were restricted, making it difficult to use them for signal collection in daily activities. A highly integrated IMU is more suitable for wearable devices and is capable of long‐term monitoring and continuous data support. For example, Ricotti V et al.^[^ ^68^ ^]^ used motion capture suits and artificial intelligence to extract digital biomarkers (Figure 3b) reflecting muscle atrophy from the daily activities of DMD patients. And the patients’ daily activities were not restricted during the data acquisition process. Although IMU home monitoring is feasible, the data they generate is enormous and complex, so more computing power and advanced algorithms are required.

In addition to IMU, conventional flexible sensors based on impedance (resistance, capacitance, or inductance) changes are also used to monitor muscle status. Structured metal materials attached to polymer films are widely used in wearable sensing and have achieved some success in health monitoring.^[^ ^69^, ^70^ ^]^ However, conventional solid metals limit such sensors’ stretchability, durability, and flexibility, so their application in soft tissues such as muscles needs to be improved. Esposito D et al.^[^ ^71^ ^]^ used dome‐structured acrylic resin (Figure 3c) to realize a piezoresistive sensor for measuring muscle contraction and mechanomyography. They demonstrated excellent sensing performance to detect tiny vibrations occurring during muscle contraction. However, the sensing material is a flexible conductive polymer, the substrate material is rigid, so the sensor they designed is not breathable and has limited wearability. Flexible sensors based on fibers/textiles can overcome the above shortcomings. Luo Heng et al.^[^ ^72^ ^]^ successfully detected the strength of lower limb skeletal muscles by using capacitive‐based flexible sensors (Figure 3d) prepared using conductive fabrics and PDMS&CB (carbon black). The sensors above focus on dynamic characteristics (pressure, velocity, acceleration, or activity space) and ignore the static characteristics of muscles, which limits their application scenarios. The change of the medium inside the inductor will be directly reflected in the sensing signal. The reduction of muscle volume corresponds to the change of the medium of the inductor sensor. For example, Rice A et al.^[^ ^73^ ^]^ used Faraday's law of induction to prepare inductive sensors on textiles (Figure 3e), which can detect changes in muscle volume under static conditions. As essential muscle signal monitoring tools, resistance, capacitance, and inductance sensors have shown broad application potential in assessing muscle atrophy. However, their self‐adhesion, sensitivity, and durability still need further in‐depth research. Therefore, future research should focus on optimizing sensor design, improving measurement accuracy, and exploring the combined use of multiple sensors to provide a more comprehensive muscle atrophy monitoring solution.

With the miniaturization of wearable devices and the demand for integrated diagnosis and treatment, more self‐powered (triboelectricity, thermoelectricity, piezoelectricity, magnetoelectricity, etc.) or highly integrated devices/systems are being developed. The explosive growth of self‐powered sensors can extend the system's standby time and avoid system redundancy.^[^ ^42^, ^74^, ^75^, ^76^ ^]^ Kong Lingji et al.^[^ ^77^ ^]^ used the principle of triboelectricity to design a self‐powered and self‐sensing lower limb system (Figure 3f). They combined it with an extended short‐term memory model to achieve the detection and supervision of Parkinson's rehabilitation status. However, this complex system focuses on detecting joint movement angles rather than direct muscle parameter measurement. Alluri NR et al.^[^ ^78^ ^]^ designed a self‐powered muscle monitoring system using the piezoelectric principle (Figure 3g). The device has a simple structure and excellent flexibility. Dong Wang et al.^[^ ^79^ ^]^ prepared an elastomer with piezoelectric properties composed of composite materials (lactic acid, butanediol, sebacic acid, and itaconic acid, Figure 3h), which achieved simultaneous repair and functional monitoring of the tibialis anterior muscle. However, such devices require more efficient energy conversion to improve operating time and reliability. And their combination with other sensing technologies (such as EMG, electrochemistry, US, etc.) can provide more comprehensive data on muscle atrophy. In addition to the mechanical sensors mentioned, we summarize the material composition, working principle, sensitivity, and extracted signal characteristics of similar sensors in detail in **Table** **2**.

**Table 2:** Material composition, working principle, sensitivity, and extracted signal characteristics of mechanical sensors for muscle atrophy detection.

Materials/Devices	Principle	Sensitivity	Signal features	Refs.
**ActiMyo**	IMU	–	Rotation rate, ratio of the vertical component in the overall acceleration, hand elevation rate, an estimate of the power of the upper limb	[67]
**Xsens suit**	IMU	–	KineDMD ethomic biomarker	[68]
**US system** +**LPMS‐ME1**	US+IMU	–	Joint angle	[80]
**VINNAPASS/ CNFs**	Piezoresistive	Max. strain: >170%	Relative resistance	[81]
**Force sensing resistors**	Piezoresistive	–	Mean value, variance, and the standard error	[82]
**Acrylic resin**	Piezoresistive	Max. press 1.6 kg	Mechanical force	[71]
**TPU/PDMS/CN**	Capacitive	0–0.2 kPa: 3.44 kPa^−1^, 0.2–2 kPa: 0.38 kPa^−1^, 2–9 kPa: 0.19 kPa^−1^	Relative capacitance	[72]
**POMaC/PGS/PLLA/Mg**	Capacitive	Min. strains: 0.4%, Min. pressures: 12 Pa	–	[83]
**40‐Filament Liberator e‐thread**	Faraday's law of induction	Muscle volume loss: 0.17 dB and 1.58° per 1% volume loss muscle circumference: 0.75 dB and 6.7° per centimeter	Magnitude and phase of the transmission coefficient	[73]
**EGaIn@silicone tubing**	Inductive loop	Magnitude and phase resolution of 1.25 dB and 3.8°, respectively	Magnitude and phase of the transmission coefficient	[84]
**Self‐sensing lower‐limb system**	Triboelectric	–	Gait characteristics	[77]
**HS‐FPCSs**	Piezoelectric	130 V/0.8 µA	Peak	[78]
**VMLRE**	Piezoelectric	–	Pulse piezoelectric peak	[79]
**AM /BIS/β‐CD PEDOT:PSS/LiCl**	Piezoelectric	Min. strains: 3%, Min. press: 0.02 N	Peak voltage	[85]
**Ecoflex and polyester/compliant silver/PVDF**	Pneumatic‐piezoelectric	−0.12 V kPa^−1^ at 50 kPa	Pulse piezoelectric peak	[86]
**PI/Ag/BaTiO_3_**	Piezoelectric	−7.79572 pC deg^−1^	Output charge	[87]

### 3.2 Electromyography

**Figure 4:**
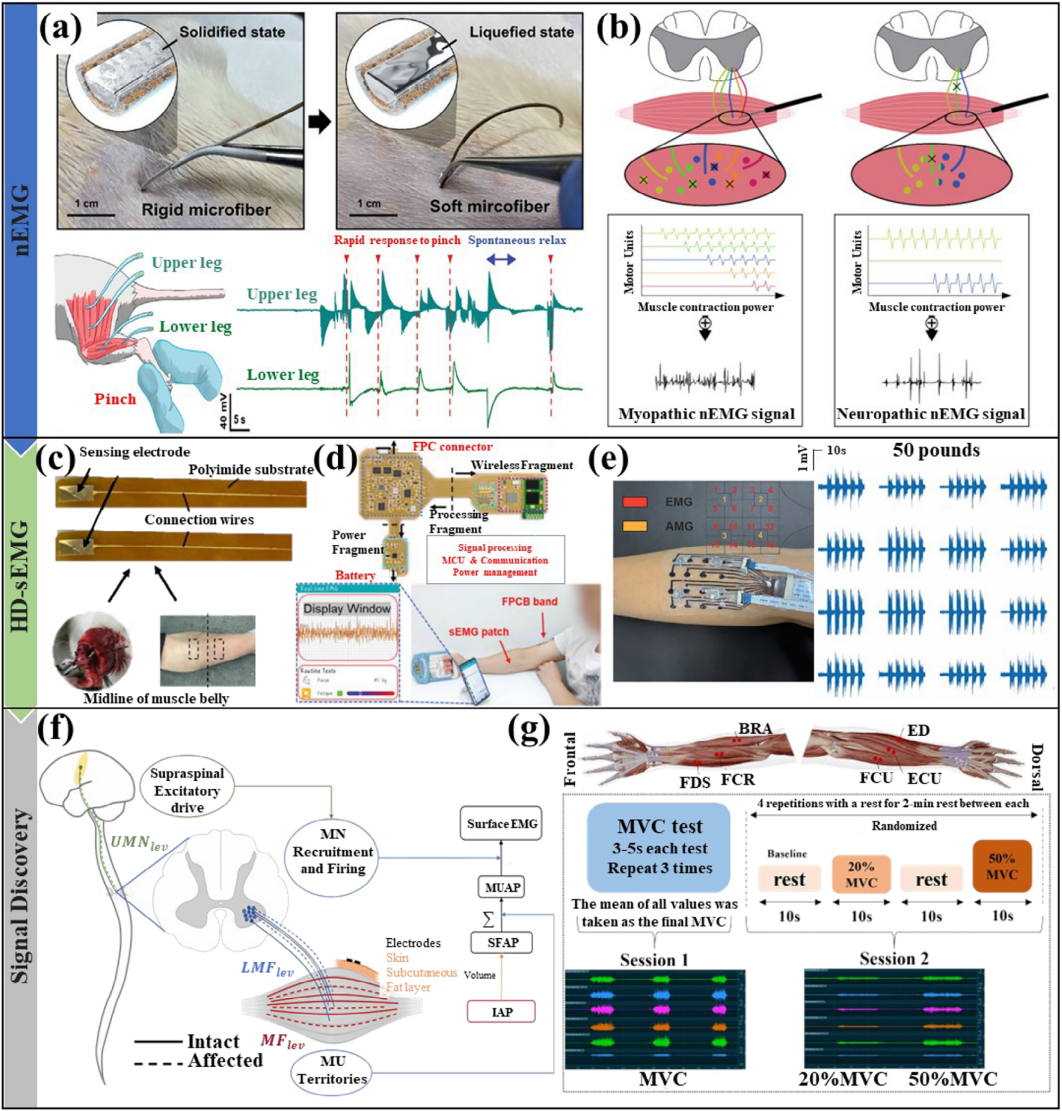
a) Optical images of biphasic ultrafine needle electrodes and EMG signals of the biceps femoris and gastrocnemius muscles. Reproduced with permission.^[^ ^95^ ^]^ Copyright 2024, Wiley. b) Schematic diagram of nEMG implantation and the electrical signals obtained for neuromuscular disease types. Reproduced with permission.^[^ ^97^ ^]^ Copyright 2022, Elsevier. c) Non‐invasive metal sEMG electrodes. Reproduced with permission.^[^ ^106^ ^]^ Copyright 2022, MDPI. d) Example of real‐time EMG data test using three electrodes. Reproduced with permission.^[^ ^107^ ^]^ Copyright 2023, Wiley. e) Piezoresistive sensors are used for EMG monitoring in situ. Reproduced with permission.^[^ ^108^ ^]^ Copyright 2024, Wiley. f) Block diagram of components and methods used to generate sEMG digital models. Reproduced with permission.^[^ ^113^ ^]^ Copyright 2023, IOPscience. g) Schematic diagram of electrode placement and sEMG signal machine learning analysis scheme. Reproduced with permission.^[^ ^96^ ^]^ Copyright 2024, BMC.

The mechanical sensors we classify mainly monitor muscle acceleration, muscle strength, or muscle volume. When muscles move, they receive electrical signals from neurons, which can be used to determine the state of nerves or muscles. The EMG signals can be divided into needle electromyography (nEMG) and surface electromyography (sEMG), depending on whether the electrodes are implanted in the body. The International Federation of Clinical Neurophysiology has issued clinical guidelines for nEMG.^[^ ^88^ ^]^ Early‐developed and highly sensitive nEMG has been widely used to diagnose various neuromuscular diseases, such as myasthenia gravis^[^ ^89^ ^]^ and ALS.^[^ ^90^, ^91^ ^]^ Hard and thick implanted electrodes cannot fit well with muscle tissue and may cause wound infection and reduce the signal‐to‐noise ratio.^[^ ^92^, ^93^, ^94^ ^]^ Flexible electrodes (such as PEDOT:PSS, PPy, Galinstan) can reduce the above problems to a certain extent. Nam Seonghyeon et al.^[^ ^95^ ^]^ designed a phase‐changeable ultrafine fiber (**Figure** **4**a) using eutectic gallium–indium and achieved electromyographic recording during leg pinching in rats. However, they did not conduct an in‐depth analysis of these signals or study the electromyographic signals associated with muscle atrophy characteristics. Since there are many causes of muscle atrophy, effective identification of electromyographic signals is an urgent problem that needs to be solved.^[^ ^96^, ^97^, ^98^ ^]^ The combination of nEMG, which more precisely captures the electrical activity of individual muscle fibers, and machine learning offers a solution for distinguishing between myopathic and neuropathic signals,^[^ ^97^ ^]^ as shown in Figure 4b. Although nEMG has achieved some success in monitoring the early stages of muscle atrophy, its invasiveness and operational complexity make it not the first choice for home monitoring of the disease.

The sEMG has better wearable characteristics and is easy to operate, so it is more widely studied in muscle atrophy monitoring.^[^ ^99^, ^100^, ^101^, ^102^, ^103^ ^]^ To analyze complex muscle atrophy states, the clarity, accuracy, and spatial resolution of sEMG signals need to be improved. Therefore, the technology has developed from simple single electrodes to large‐area electrode arrays.^[^ ^104^, ^105^ ^]^ Figure 4c shows the bimetallic flexible electrode Pei Xiachuan et al.^[^ ^106^ ^]^ used to distinguish muscle atrophy caused by disuse and nerve damage. But its Au electrodes are not stretchable, have no antibacterial properties, and the collection of electrical signals relies on a laptop. Gong Qibei et al.^[^ ^107^ ^]^ designed a more flexible three‐electrode (Au mesh) system equipped with a flexible circuit board to achieve real‐time monitoring of muscle strength and fatigue, as shown in Figure 4d. However, the limited channels make the available muscle signals very limited, which is not conducive to judging the muscle status of a large area. Li Tengfei et al.^[^ ^108^ ^]^ used a stretchable sensor (Figure 4e) with 16‐channel electrodes (liquid metal) and a 4‐channel microphone (ICS‐40300) to characterize muscle activity. The development of high‐density surface electromyography (HD‐sEMG) has made it possible to collect more useful information about muscle atrophy. As a result, there has been an explosion of methods to analyze this data.^[^ ^102^, ^109^, ^110^, ^111^, ^112^ ^]^ Li Guijin et al.^[^ ^113^ ^]^ used the components of the generated sEMG model shown in Figure 4f to analyze that changes in the number of muscle fibers would cause autoregressive and cepstral coefficients, but were limited to model calculations. Li Na et al.^[^ ^96^ ^]^ achieved the diagnosis of sarcopenia in Chinese community‐dwelling elderly people using the experimental protocol shown in Figure 4g. The signals of sEMG may be affected by skin contact, motion artifacts, and individual physiological differences. Therefore, developing effective signal processing and personalized analysis methods is particularly important for diagnosing muscle atrophy.

### 3.3 Bioelectrical Impedance

**Figure 5:**
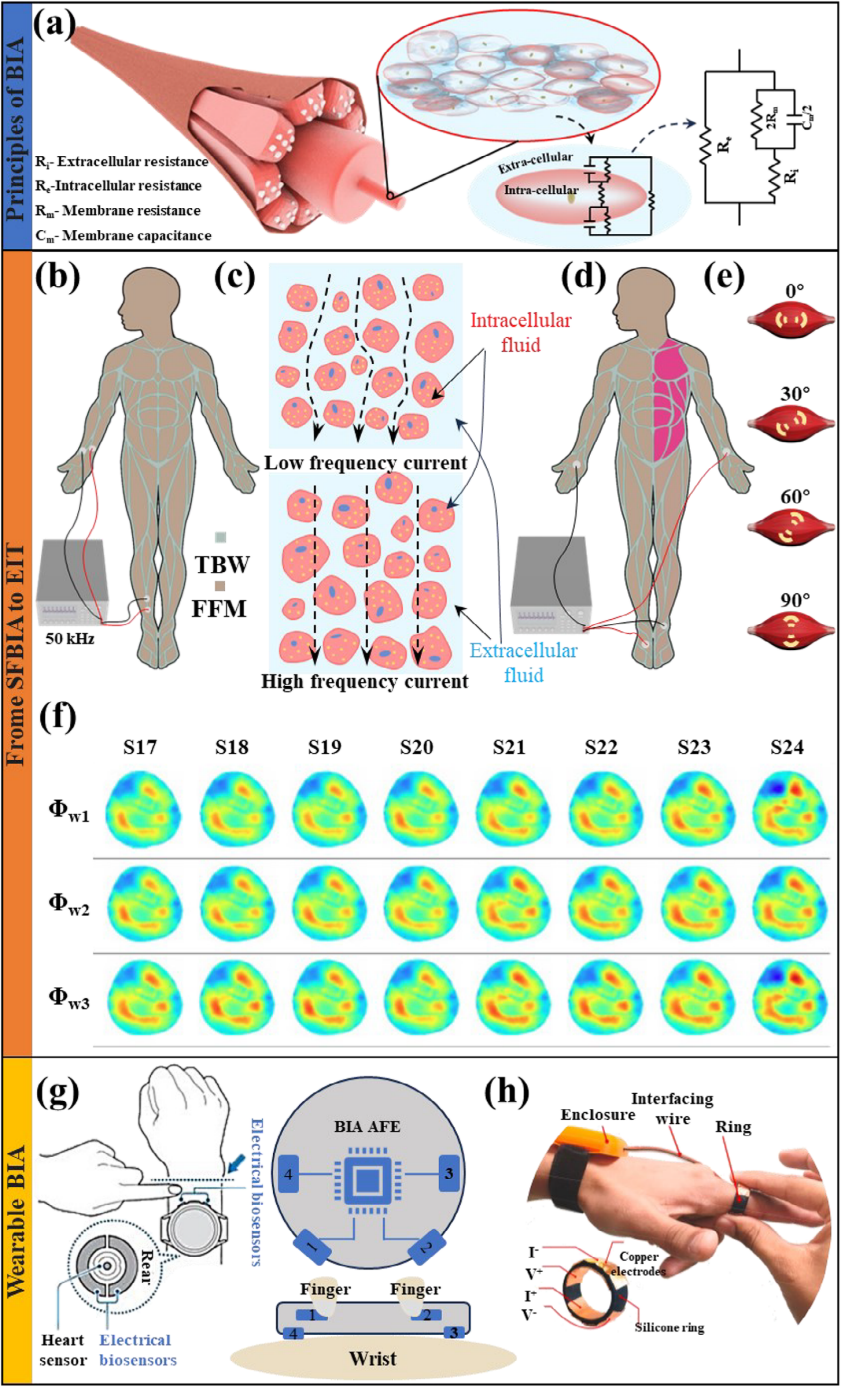
a) BIA equivalent circuit diagram obtained from the single cell body fluid environment. b) Schematic diagram of SFBIA for measuring body TBW and FFM. c) The difference between high and low frequency currents passing through muscle cells. d) Schematic diagram of MFBIA for analyzing human segmental components. e) Basic concept of rotational EIM. The entire electrode array is rotated around a central point to study the anisotropy of the muscle. f) Phase angle muscle imaging obtained by EIT in different subjects. Reproduced with permission.^[^ ^129^ ^]^ Copyright 2024, IOPscience. g) BIA‐based wearable watch for body composition analysis. Reproduced with permission.^[^ ^130^ ^]^ Copyright 2022, Elsevier. h) Ring with BIA function. Reproduced with permission.^[^ ^134^ ^]^ Copyright 2022, Elsevier.

EMG signals are only obtained when muscles move or are stimulated, and when muscle atrophy occurs, its static impedance will also change. This is because the electrical impedance comes from the resistance of the intracellular fluid, the extracellular fluid, and the membrane, as well as the capacitance on the membrane,^[^ ^114^, ^115^ ^]^ as shown in **Figure** **5**a. Therefore, BIA‐related technologies are also widely used in monitoring muscle atrophy.^[^ ^116^, ^117^ ^]^

At the beginning of the development of this technology, a single‐frequency (50 kHz) power supply was mainly used to obtain body impedance data. Still, it could only analyze Total Body Water (TBW) and Fat‐Free Mass (FFM),^[^ ^118^, ^119^, ^120^ ^]^ as shown in Figure 5b. This is because low‐frequency electrical signals can only pass through the interstitial fluid. However, as the frequency increases, the signal can pass through the cell's interior and provide richer information (such as body cell mass, phase angle, and intracellular water) about human tissues,^[^ ^114^, ^118^, ^121^, ^122^, ^123^ ^]^ as shown in Figure 5c. This is also the theoretical basis for using multi‐frequency BIA (MFBIA) to analyze some components of our body. Figure 5d shows a typical 4‐electrode configuration for left torso detection.^[^ ^124^ ^]^ Thanks to advances in sensing, computing power, and imaging algorithms, electrical impedance tomography (EIT) based on the principle of biological tissue conductivity has begun to develop rapidly.^[^ ^125^ ^]^ Initially, the linear representation of muscles was used, and then the rotational electrical impedance myography (EIM), as shown in Figure 5e, became widely used.^[^ ^119^, ^126^, ^127^, ^128^ ^]^ For example, Figure 5f shows the phase angle distribution (Ф_w1_–Ф_w5_) of different subjects (S17–S24) at the optimal voltage intensity observed by Sun Bo et al.^[^ ^129^ ^]^ using EIT. Darma Panji Nursetia et al.^[^ ^126^ ^]^ also used the phase angle of EIT to discover differences in the local muscle quality between young and middle‐aged subjects. However, the accuracy of BIA is easily affected by body fluids, and its sensitivity for early detection of muscle atrophy is insufficient. Therefore, high‐precision and reliable BIA devices must be further developed to monitor muscle status accurately.

So, although there are many reports on wearable watches^[^ ^130^, ^131^, ^132^, ^133^ ^]^ and rings^[^ ^134^ ^]^ (Figure 5g,h), most of them only focus on fat content analysis. Bennett J P et al.^[^ ^130^ ^]^ compared the data obtained from commercial watches with DXA. They found that although there were differences in accuracy, the trends were consistent and could be used to improve patients' quality of life. In addition to the miniaturization of hardware, advances in theoretical knowledge^[^ ^135^ ^]^ and computational models^[^ ^136^, ^137^ ^]^ related to bioelectrical impedance have made more detailed local EIM feasible.^[^ ^138^, ^139^ ^]^ These all support the future application of wearable EIM for the early detection of muscle atrophy.

### 3.4 Ultrasound Patch

**Figure 6:**
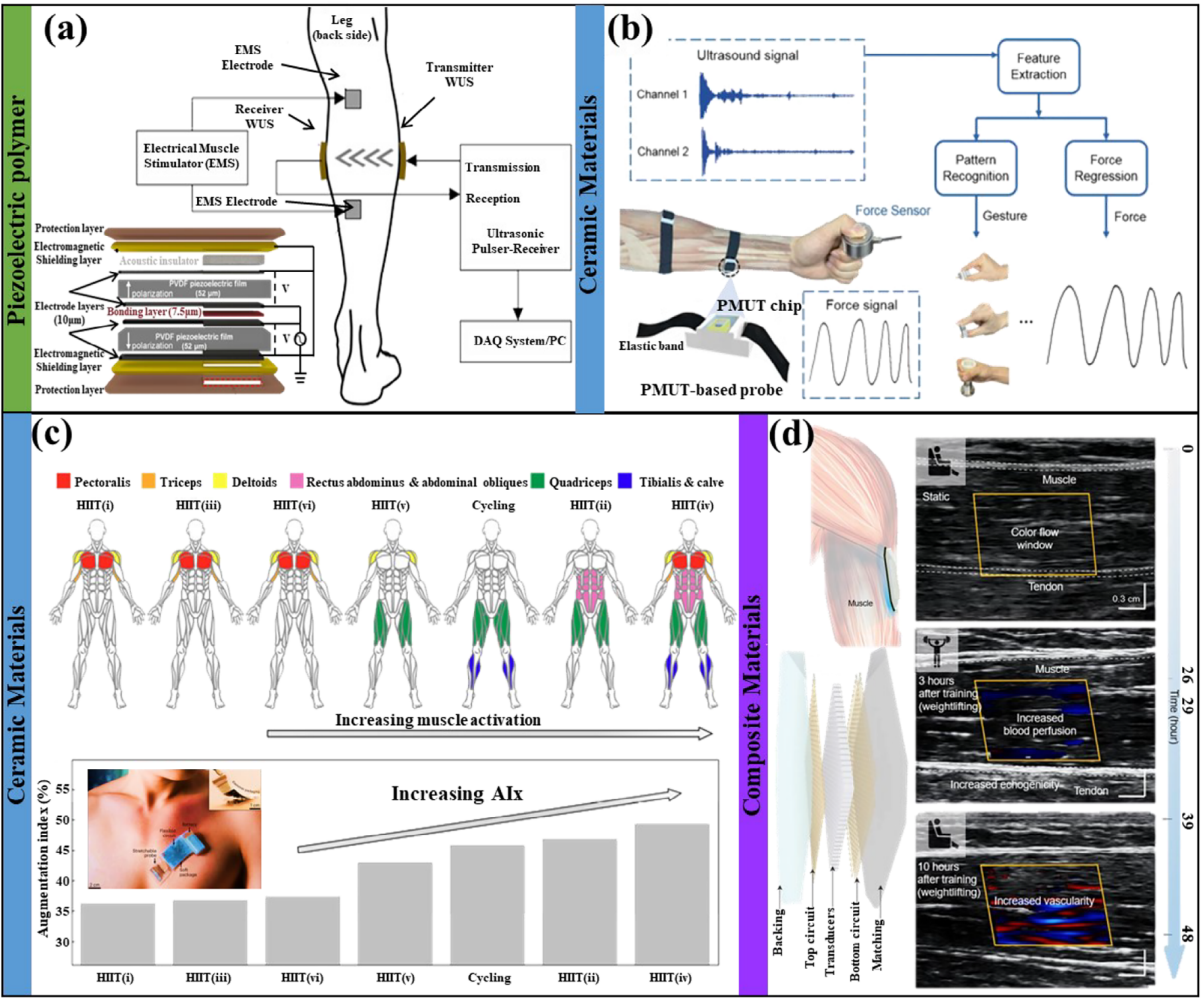
a) Ultrasonic testing protocol for measuring skeletal muscle contraction parameters. Reproduced with permission.^[^ ^149^ ^]^ Copyright 2020, MDPI. b) Piezoelectric micromachined ultrasonic transducer for muscle force estimation. Reproduced with permission.^[^ ^154^ ^]^ Copyright 2024, Elsevier. c) Wearable US system monitors muscle recruitment and corresponding augmentation index during cycling and high‐intensity interval training. Reproduced with permission.^[^ ^155^ ^]^ Copyright 2023, Springer Nature. d) US imaging of the biceps brachii at different periods before and after weightlifting training. Reproduced with permission.^[^ ^156^ ^]^ Copyright 2022, Science.

Muscle US relies on the reflection of sound waves to obtain signals. A transducer transmits high‐frequency sound waves into the body, which are reflected when they encounter the boundaries between different tissues (such as muscle and bone). The reflected sound waves are converted into electrical signals, which show the structure of the muscle and any abnormalities. It has long been proven to help monitor muscle atrophy,^[^ ^140^, ^141^, ^142^, ^143^, ^144^ ^]^ but floor‐standing or handheld devices are bulky, complex to maintain, and require professional assistance. With the emergence of new ultrasonic transducer materials and the advancement of micro‐nan technology,^[^ ^145^, ^146^, ^147^, ^148^ ^]^ ultrasonic devices are constantly miniaturized to meet the needs of wearable devices. Materials that can be used for flexible ultrasonic transducers include piezoelectric polymers (such as polyvinylidene difluoride, polyacrylonitrile), piezoelectric ceramics (calcium titanate, zinc oxide, lead zirconate titanate (PZT), and hydroxyapatite), single crystal materials (LiNbO3, lead‐magnesium niobate lead‐titanate (PMN‐PT)), and composite materials. Ultrasonic transducers made of piezoelectric polymers have the advantages of good flexibility and low acoustic impedance and are used to monitor the static and dynamic parameters of skeletal muscles.^[^ ^149^, ^150^, ^151^, ^152^ ^]^ For example, AlMohimeed I et al.^[^ ^149^ ^]^ measured the tissue thickness of the gastrocnemius muscle under ES using the system shown in **Figure** **6**a.

Piezoelectric ceramic materials with wide‐band response characteristics and easy production are used in muscle strength, fatigue, vasodilation, and blood flow in muscle groups.^[^ ^153^, ^154^, ^155^ ^]^ Qu Mengjiao et al.^[^ ^154^ ^]^ used the aluminum nitride‐based piezoelectric micromachined ultrasonic transducer shown in Figure 6b to achieve gesture recognition and successfully estimate muscle strength. However, the sensor was ultimately encapsulated by a plastic shell, which would affect the comfort of wearing to a certain extent. Therefore, a fully flexible PZT‐based US system (Figure 6c) was developed and implemented to calculate the vascular enhancement index in active muscles before and after exercise.^[^ ^155^ ^]^ Although ceramic materials are brittle and have poor stability, miniaturizing them and encapsulating them with flexible materials can significantly improve their durability and flexibility.^[^ ^148^ ^]^ For example, Wang Chonghe et al.^[^ ^156^ ^]^ firmly combined an US probe with a coupling agent made of a hydrogel‐elastomer mixture (Figure 6d) to achieve changes in blood perfusion, echo changes, and vascular distribution before and after weightlifting training. Compared with traditional ultrasonic equipment, the signals of patch‐type ultrasonic devices are more susceptible to interference from external noise, resulting in poor imaging effects and data quality. The technology is currently in its initial exploratory stage, and there have been no reports of large‐scale preparations, and the interpretation of its data is not standardized enough.

### 3.5 Electrochemical Sensors

When muscle atrophy occurs, in addition to changes in its physical parameters, the physiological indicators of the human body will also change accordingly. Electrochemical sensors can quantitatively detect blood, interstitial fluid (XA), and sweat biomarkers.^[^ ^31^, ^157^, ^158^ ^]^ They work by exploiting the electrical activity generated by the chemical reaction between the muscle atrophy biomarker and the sensing electrode. This electrical activity, usually in the form of current or voltage, is converted into a measurable signal proportional to the analyte concentration. Currently, the biomarkers that are directly related to muscle atrophy include the following types of enzymes (creatine kinase, superoxide dismutase), proteins (myoglobin, tumor necrosis factor, cardiac myoglobin), metabolites (creatinine), and hormones (hydrocortisone, insulin‐like growth factor 1).^[^ ^159^, ^160^, ^161^ ^]^ Among them, creatinine is the most widely studied because it is easy to extract and exists in blood (44.21–106.10 µm), urine, and interstitial fluid.^[^ ^162^, ^163^ ^]^ Enzymatic reaction is one of the classic ways to prepare electrochemical sensors, and creatinine detection is no exception.^[^ ^164^ ^]^ Although this type of sensor has high selectivity and good sensitivity, it is easily affected by factors such as ambient temperature, pH value, and ionic strength. The extraction cost is also high, and it is challenging to preserve. Currently, non‐enzymatic creatinine detection sensors mainly rely on the reduction reaction of Cu^2+^ and creatinine.^[^ ^163^, ^165^, ^166^ ^]^ This dramatically reduces the preparation cost of creatinine sensors. However, factors that can cause changes in creatinine concentration include muscle atrophy and impaired kidney function.^[^ ^162^, ^167^, ^168^ ^]^ Screen‐printed sensors for detecting survival motor neurons that are more specific for SMA‐induced muscle atrophy were developed.^[^ ^169^ ^]^ In addition, multi‐sensor integrated devices for muscle atrophy markers have also been designed. For example, Eissa Shimaa et al.^[^ ^170^ ^]^ designed a carbon nanofiber‐based multiplex immunosensor to simultaneously detect the respective biomarkers SMN1 and DMD protein of SMA and DMD. More information about electrochemical sensors for muscle atrophy biomarkers is listed in **Table** **3** for comparison. This type of sensor has high sensitivity and is suitable for the early detection of muscle atrophy. However, since most of the parameters detected are biochemical, sampling is invasive, and the samples are easily contaminated.

**Table 3:** Comparison of electrochemical sensors for the detection of different muscle atrophy biomarkers.

Materials	Biomarkers	Sensitivity/detection limits (DL)	Refs.
**PS/V–TiO_2_**	Creatinine	30.95 ± 1.73 µA cm^−2^ µm ^−1^	[162]
**Cu/SPCE**	Creatinine	DL: 0.0746 µm	[163]
**BNNTs/CNN/CRN/SOX **	Creatinine	72 µA (mg^−1^ dL^−1^)	[164]
**Cu^2+^/Pt**	Creatinine	5401 ± 99 A m^−2^ m ^−1^	[166]
**PMB‐PVAc‐Cu‐CNF/ACF**	Creatinine	0.133 µA ng mL^−1^	[168]
**Carbon nanomaterial**	Survival motor neuron	DL: 0.75 pg mL^−1^	[169]
**ssDNA/Sm_2_O_3_ NPs‐rGO/PANI/GCE**	SOD1	DL: 1 × 10–14 mol L^−1^	[171]
**Poly‐*o*‐PD/MIP layer**	Cytochrome c	9.8 × 1011 µA m ^−1^	[172]
**AuNP‐Fe_2_O_3_NC and MB/[Fe(CN)_6_]^3−^**	miR‐338‐3p	–	[173]
**MnO@CNTs/GCE**	Cardiac myoglobin	0.88994 µA µg^−1^ mL^−1^	[174]
**IL‐6 and TNF‐α antibodies**	IL‐6 and TNF‐α	–	[175]
**Antibodies/BSA/ CNF**	CFTR, DMD, SMN1	DL: 0.9, 0.7, 0.74 pg mL^−1^	[170]

## 4 Treatment

In addition to detecting muscle atrophy, wearable devices can achieve effective prevention and treatment. This is mainly due to the following aspects: 1) Powered exoskeletons can effectively enhance athletic endurance and strength and provide sufficient support for atrophied muscles.^[^ ^176^, ^177^, ^178^ ^]^ Their increasingly lightweight and flexible design makes daily wear a reality. 2) A range of physical modalities^[^ ^179^, ^180^, ^181^, ^182^, ^183^, ^184^, ^185^ ^]^ that are effective in treating muscle atrophy (e.g., electromagnetic stimulation, exercise) can be achieved using simple, portable actuators. 3) The diversity of power sources^[^ ^74^, ^186^ ^]^ (triboelectric nanogenerator, piezoelectric nanogenerator, and biofuel cell) provides a safer and more efficient way for ES and drug release. We divide them into four categories (transcutaneous electrotherapy, bioelectronic implants, bioelectronic drug delivery, and ExoMuscles) according to their working principles and action sites for separate reviews.

### 4.1 Transcutaneous Electrotherapy

Transcutaneous electrotherapy delivers the electric current to the skin's surface through electrodes to activate nerves and muscles. This stimulation can trigger a series of physiological reactions, including muscle contraction, reduced capillary regression,^[^ ^187^ ^]^ increased protein expression or absorption^[^ ^188^, ^189^, ^190^ ^]^ (skeletal muscle protein, protein‐derived amino acids), and improved nerve conduction.^[^ ^191^ ^]^ Although the effectiveness of ES in preventing and alleviating muscle atrophy was discovered as early as the 1940s, there is no unified understanding of the mechanism. However, related exploration has never stopped due to its non‐invasive, low‐cost, and easy access characteristics. The main research issues can be divided into two aspects: one is to select the most appropriate electrical signal, and the other is to choose the electrical release location.

**Figure 7:**
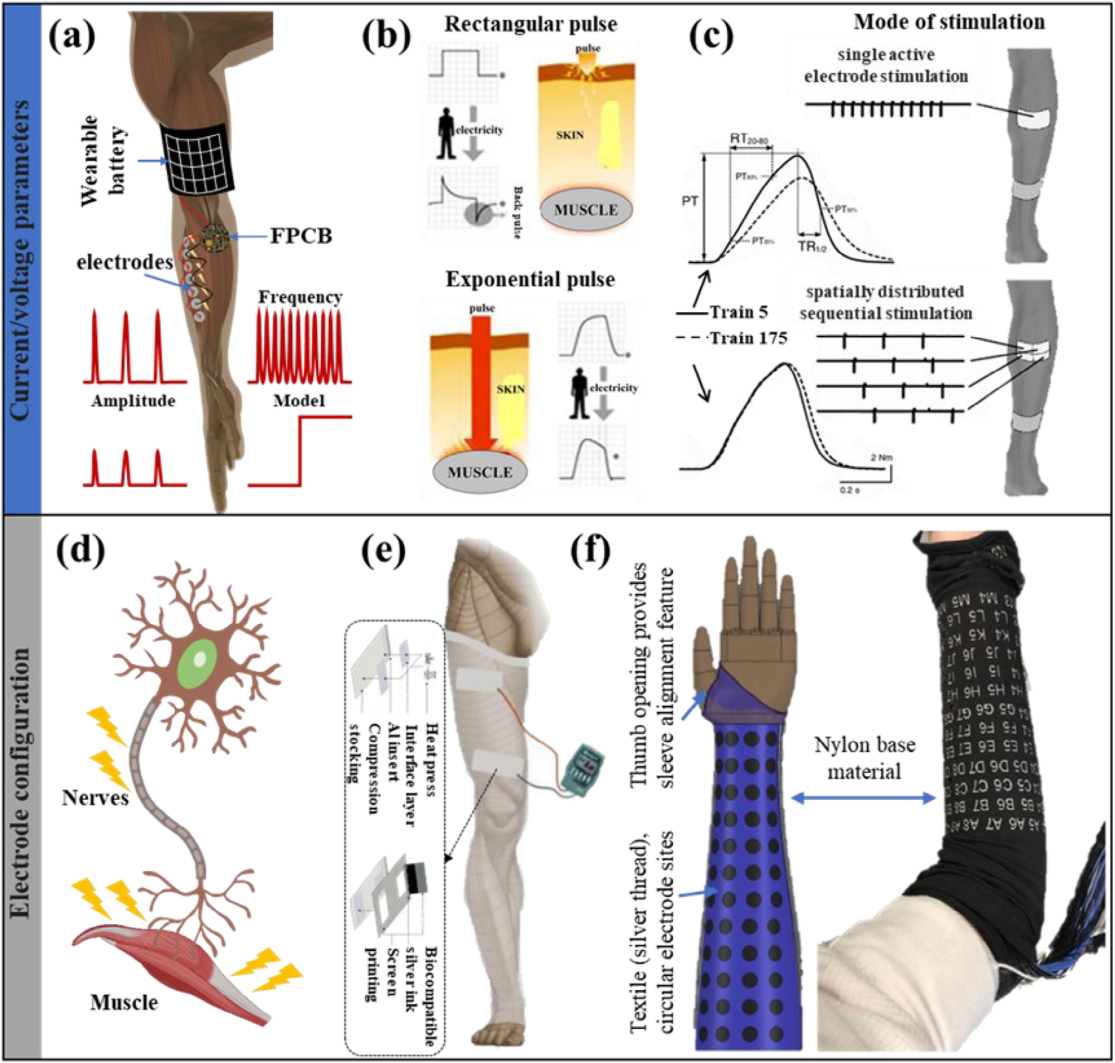
a) Schematic diagram of wearable electronic transcutaneous ES for muscle atrophy and examples of variable parameters. b) Comparison of rectangular pulse and exponentially rising pulse. Reproduced with permission.^[^ ^199^ ^]^ Copyright 2011, Elsevier. c) Schematic diagram of spatially and sequentially distributed power for transcutaneous ES. Reproduced with permission.^[^ ^204^ ^]^ Copyright 2014, Springer Nature. d) Schematic diagram of the optional sites for neuromuscular ES. e) Anti‐embolism stockings with dual electrodes are used to reduce muscle atrophy in the vastus lateralis. Reproduced with permission.^[^ ^216^ ^]^ Copyright 2012, RSC. f) Multi‐electrode array modulates patient grip strength. Reproduced with permission.^[^ ^218^ ^]^ Copyright 2019, Springer Nature.

Research on high‐quality electrical signal selection mainly focuses on signal frequency, amplitude, waveform, time, etc.^[^ ^192^, ^193^, ^194^, ^195^, ^196^ ^]^ Advances in microelectronics technology have made it easy to condition electrical signals in wearable systems, as shown in **Figure** **7**a. Short pulse electrical signals are suitable for preventing and reducing muscle atrophy, while continuous ES is more suitable for pain management, so pulse signals are our focus. The amplitude and duration of electrical signals are directly related to human health, because excessive current can cause shock or even death,^[^ ^197^ ^]^ and prolonged ES can easily burn the skin.^[^ ^198^ ^]^ Regarding waveform selection, there is evidence that exponential climbing pulse waves are more effective in reducing discomfort during muscle stimulation,^[^ ^199^, ^200^ ^]^ as shown in Figure 7b. Bellew J W et al.^[^ ^201^ ^]^ showed that interfering and burst‐modulated biphasic pulse current produced greater muscle force than Russian current when phase duration, frequency, and amplitude were similar. Studies of pulse frequency have found that low frequencies (<30 Hz) have limited therapeutic effects, while high frequencies (30–100 Hz) have deeper penetration but cause stinging.^[^ ^195^, ^202^, ^203^ ^]^ To this end, interesting spatial and sequentially distributed ES (Figure 7c) schemes were designed,^[^ ^204^ ^]^ which evenly distributed high‐frequency ES to multiple nearby electrodes. In addition, discussions about pulse width and waveform are also common.^[^ ^24^, ^195^, ^198^, ^205^ ^]^

In addition to the many options for the characteristics of ES itself, the location and number of electrode sites also significantly impact the treatment effect.^[^ ^206^, ^207^, ^208^ ^]^ Previous studies have shown that muscle strength and size correlate with the neuromuscular junction.^[^ ^209^, ^210^ ^]^ Transcutaneous electrical nerve stimulation usually simultaneously acts on nerve endings and muscle tissue (Figure 7d). Still, the coverage area and distribution of the electric field have noticeable differences in the stimulation effect. Therefore, the configuration of electrodes is also one of the key difficulties in research.^[^ ^206^, ^208^, ^211^ ^]^ Electric fields have been shown to promote myoblast proliferation, myoblast‐myotube fusion, and myosin heavy chain expression^[^ ^212^, ^213^, ^214^ ^]^ in skeletal muscle tissue engineering. Dual‐electrode ES (Figure 7e) is a common electrode configuration that can effectively stimulate target muscles and increase the conduction of nerve signals.^[^ ^215^, ^216^, ^217^ ^]^ The electrode/tissue interface charge injection mechanism shows that a larger electrode surface area helps alleviate overpotential.^[^ ^205^ ^]^ Finite element simulation results show that a reasonable electrode spacing helps reduce current loss.^[^ ^208^ ^]^ Ciancibello John et al.^[^ ^218^ ^]^ used the electrode layout shown in Figure 7f to achieve feedback stimulation of muscle grip force. This high‐density electrode stimulation method can make ES more uniform. However, it affects the patient's daily activities, easily causes muscle fatigue, and increases electrical risks.

### 4.2 Bioelectronic Implants

**Figure 8:**
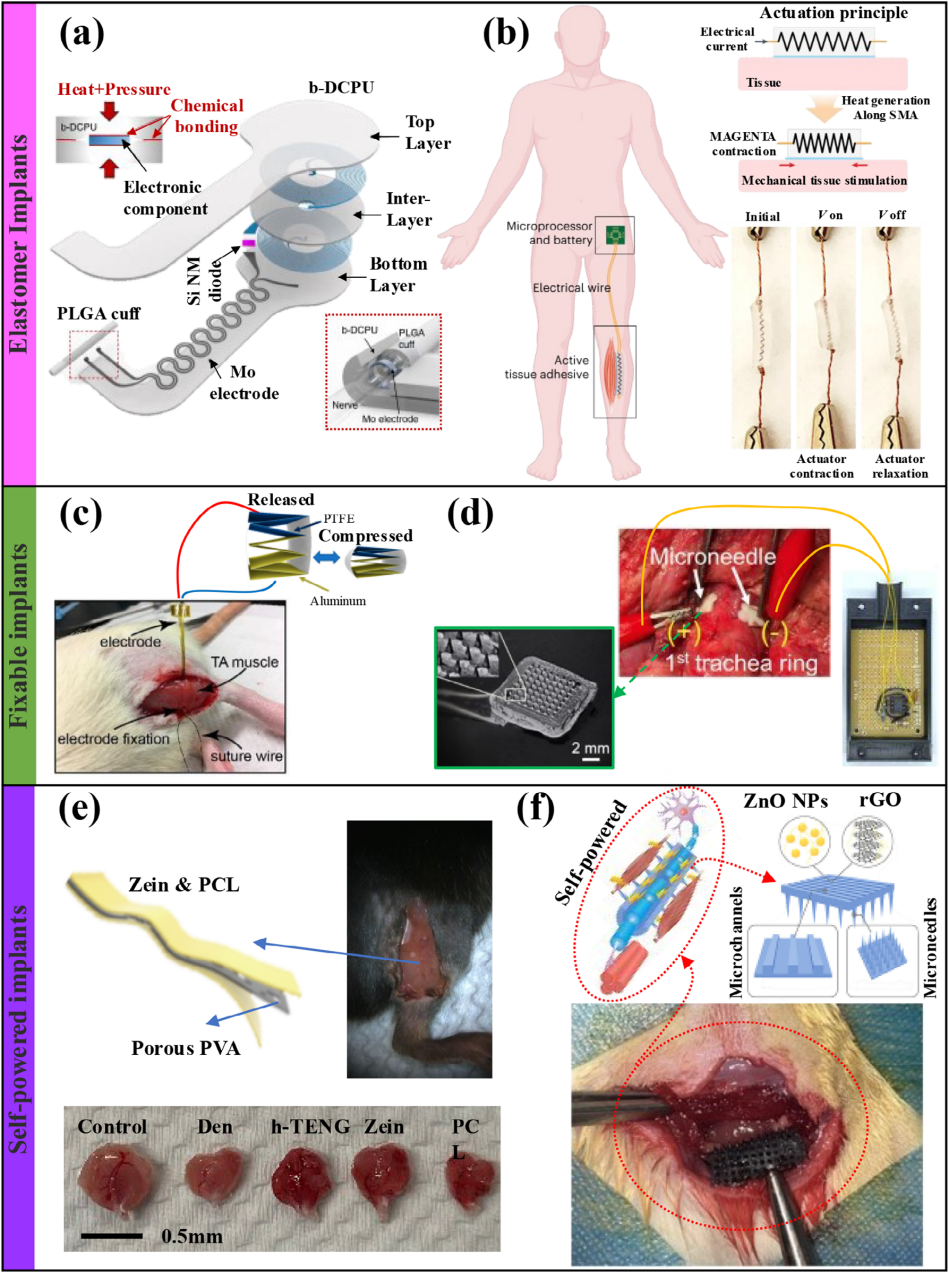
a) Stretchable, dynamic covalent polymer‐based electronic stimulators for neuromuscular regeneration. Reproduced with permission.^[^ ^226^ ^]^ Copyright 2020, Springer Nature. b) Shape memory alloy‐driven elastomeric adhesive reduces muscle atrophy. Reproduced with permission.^[^ ^229^ ^]^ Copyright 2022, Springer Nature. c) Schematic diagram of a triboelectric nanogenerator integrated with sutured intramuscular electrodes for direct muscle stimulation. Reproduced with permission.^[^ ^230^ ^]^ Copyright 2019, ACS. d) MXene nanosheet‐based microneedles for electrical muscle stimulation therapy. Reproduced with permission.^[^ ^222^ ^]^ Copyright 2021, ACS. e) Fully implantable triboelectric nanogenerator for prevention of denervated muscle atrophy. Reproduced with permission.^[^ ^9^ ^]^ Copyright 2024, Elsevier. f) Self‐powered microneedle nerve conduit for muscle atrophy inhibition and promotion of nerve repair. Reproduced with permission.^[^ ^224^ ^]^ Copyright 2024, ACS.

Although percutaneous ES can effectively prevent or alleviate muscle atrophy. However, its penetration depth is limited,^[^ ^195^ ^]^ the electrodes need to be replaced frequently, and it cannot directly act on the diseased muscles. Implantable ES can effectively avoid the above shortcomings and achieve more efficient treatment of muscle atrophy.^[^ ^195^, ^219^ ^]^ Implantable bioelectronics can be divided into semi‐implantable and fully implantable categories. The former has interfaces for external devices,^[^ ^220^, ^221^, ^222^, ^223^ ^]^ while the latter has no interfaces and is mostly self‐powered^[^ ^9^, ^224^, ^225^ ^]^ or wirelessly connected.^[^ ^226^ ^]^ The implant site is subject to the stretching of daily muscle activity, so maintaining a stable electrode–tissue interface is critical.^[^ ^227^ ^]^ Materials that match the mechanical properties of muscle tissue can be used to achieve coordinated movement of the muscle and the device. Elastomer materials have been proven to have the abovementioned properties and are widely used in implantable electrode materials.^[^ ^228^ ^]^ For example, Choi Yeon Sik et al.^[^ ^226^ ^]^ designed a stretchable, micro‐expandable implantable electronic stimulator made of dynamic covalent polyurethane material as shown in **Figure** **8**a. And they successfully used the device to promote neuromuscular regeneration under ES conditions. However, the device is activated by an external transmission coil, and its efficiency is critically dependent on the distance between the coil and the body. However, the electrically controlled actuator developed by Nam Sungmin et al.^[^ ^229^ ^]^ for preventing muscle atrophy (Figure 8b) is directly activated by low‐voltage electricity. And the self‐adhesive nature of the device provides reliable adhesion for the muscle–actuator interface.

A stable electrode–tissue interface can prevent the device from falling off to achieve stable ES and avoid muscle fatigue and burns. Therefore, techniques such as sutures,^[^ ^230^ ^]^ microneedle electrodes,^[^ ^221^, ^222^, ^231^ ^]^ and adhesives enhance their stability. For example, Wang Jiahui et al.^[^ ^230^ ^]^ sutured thin film electrodes to mouse muscles to achieve stable ES, as shown in Figure 8c. Suturing of the electrodes causes muscle damage, increases the risk of tissue infection, and may lead to rejection. However, fitted microneedles can effectively reduce the above risks. Yang Yen‐Chang et al.^[^ ^222^ ^]^ used microneedles made of PLA and MXene materials and autonomous circuits (Figure 8d) to realize a portable electrical stimulator to control muscle contraction and tremor. However, the device requires an external power supply, while self‐powered devices can increase portability and are therefore widely used in wearable ES.^[^ ^74^, ^221^, ^227^, ^232^ ^]^ The fully implanted self‐generating device can not only avoid infection of open wounds but also simplify the usage process and provide continuous treatment effects, which provides a better solution for ES treatment of muscle atrophy. Zhang Shuai et al.^[^ ^9^ ^]^ effectively prevented muscle atrophy using implantable triboelectric nanogenerators (Figure 8e) and discovered heterogeneous structures in negative friction materials. This device's fixation depends on zein's gelatinization properties, but the reliability is also limited. Hu Cewen et al.^[^ ^224^ ^]^ used piezoelectric materials to design a fixable ES microneedle (Figure 8f) to achieve synchronous repair of nerves and muscles. In addition to the tissue–electrode interface, the structure,^[^ ^233^ ^]^ material,^[^ ^234^ ^]^ and layout of the electrodes^[^ ^235^ ^]^ and the selection of electrical signal parameters^[^ ^236^, ^237^ ^]^ are also of great concern for implant‐based treatment of muscle atrophy. However, the inherent disadvantages of implantable therapies, such as susceptibility to infection, device removal, and lack of visualization, greatly limit their scope of use.

### 4.3 Bioelectronic Drug Delivery

**Figure 9:**
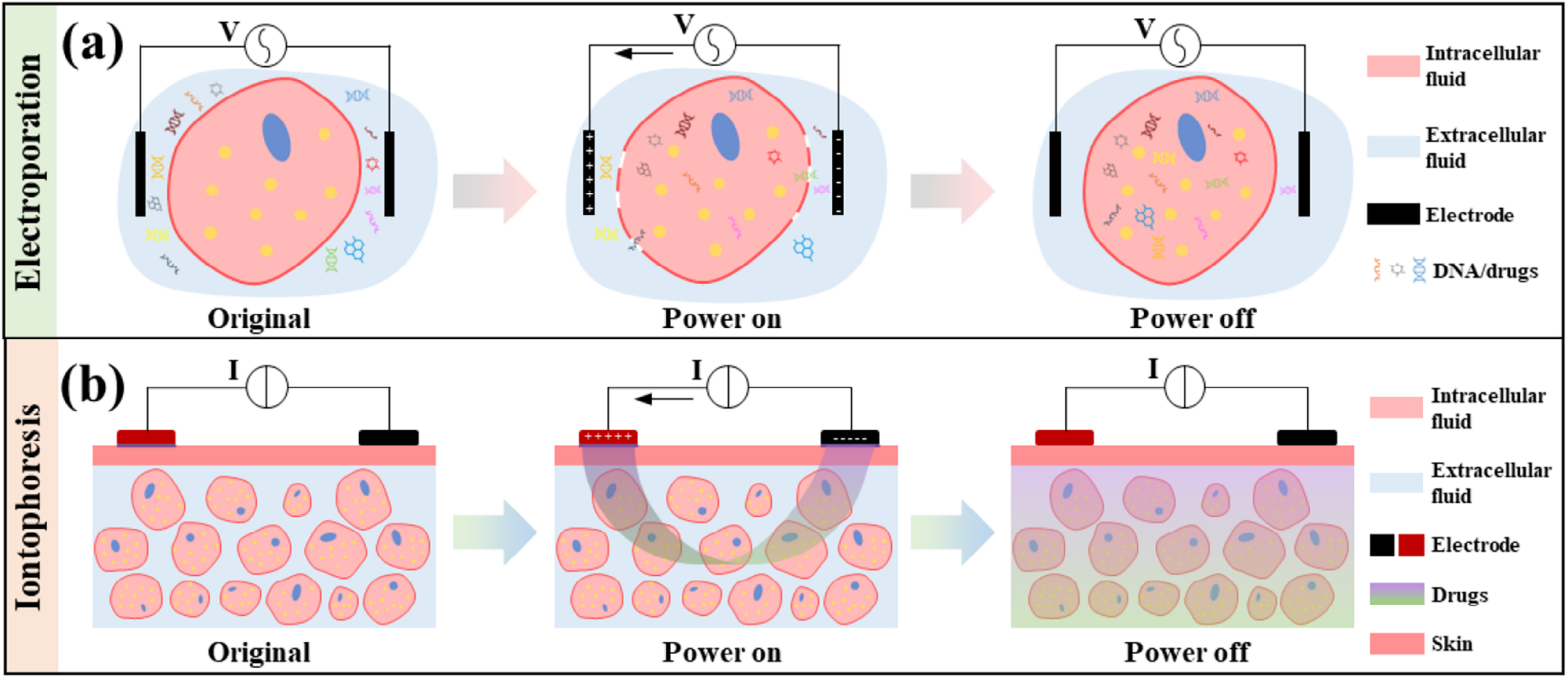
Schematic diagram of a) how electroporation and b) iontophoresis work.

In addition to ES inside the body, which can effectively prevent and alleviate muscle atrophy, drugs^[^ ^44^, ^238^, ^239^ ^]^ or gene therapy^[^ ^240^, ^241^ ^]^ have also been shown to have excellent effects. To further improve the absorption rate, targeting and controllability of drugs, ES drug delivery has received increasing attention.^[^ ^242^, ^243^, ^244^, ^245^, ^246^ ^]^ The bioelectronic drug delivery system has also significantly treated muscle atrophy.^[^ ^247^, ^248^, ^249^ ^]^ Based on its working principle, it can be divided into electroporation and iontophoresis. The working principle of electroporation is shown in **Figure** **9**a. The cell membrane opens under the stimulation of high voltage (generally >100 V), and the drug (mainly DNA or RNA^[^ ^250^ ^]^) enters the cell; after the voltage stops, the cell membrane returns to its original state, and the drug immediately takes effect. Schertzer J. D. et al.^[^ ^251^ ^]^ found that electroporation gene delivery of IGF‐I protein is more effective in repairing muscles than micro‐osmotic pump delivery of IGF‐I protein. Excessively high voltages place higher demands on the duration of stimulation release and cell tolerance. In addition, the limited penetration range of electroporation and the complex physiological environment of the human body restrict its further development. Iontophoresis with a lower voltage (generally <10 V) is safer, and its working principle is shown in Figure 9b. This technology usually uses constant or pulse current, and the reduction in voltage allows small molecules or ionic drugs to pass through the original membrane channels and enter the cell. Michiue Kohki et al.^[^ ^249^ ^]^ successfully injected myostatin inhibitory‐D‐peptide‐35 (MID‐35) into the skeletal muscle of mice through the skin using iontophoresis and achieved an increase in muscle mass. This also demonstrates the potential of this therapy in delivering large molecules and provides more possibilities for bioelectronic drug release. However, this direct ES method of drug delivery exposes patients to higher electrical risks. More examples of bioelectronic drug delivery for preventing and treating muscle atrophy are listed in **Table** **4**. The combined application of various bioelectronic drug release methods with microneedles and nanocarriers provides possibilities for the prevention and treatment of various types of muscle atrophy.^[^ ^252^ ^]^

**Table 4:** Some examples of bioelectronic drug delivery for preventing and treating muscle atrophy.

Drug	Operation mode	Electrical parameters	Refs.
**Aspirin or ibuprofen**	–	Duration: 100–200 µs; voltage: 100–500 mVpp	[231]
**Plasmids CMVb**	Electroporation	175 V cm^−1^, 1 Hz	[247]
**pcDNA3‐EGFP**	Electroporation	12.5 V mm^−1^, pulse length: 20 ms, number of pulses: 5, interval: 200 ms	[248]
**IGF‐I gene**	Electroporation	Three 20 ms square wave pulses (75–100 V cm^−1^) of 1 Hz	[251]
**SIRT1 Protein**	Electroporation	–	[253]
**MID‐35**	Iontophoresis	0.34 mA cm^−2^ for 1 h	[249]
**Gold nanoparticles**	Iontophoresis	300 µA, 6 min	[254]
**Glycocol‐Mecholyl**	Iontophoresis	2 mA for 6–7 h or 20 mA for 3–5 h	[255]
**GFP‐C2C12 myoblasts**	–	–	[256]
**Gelatin–Polyaniline nanofibers**	–	–	[257]

### 4.4 ExoMuscles

**Figure 10:**
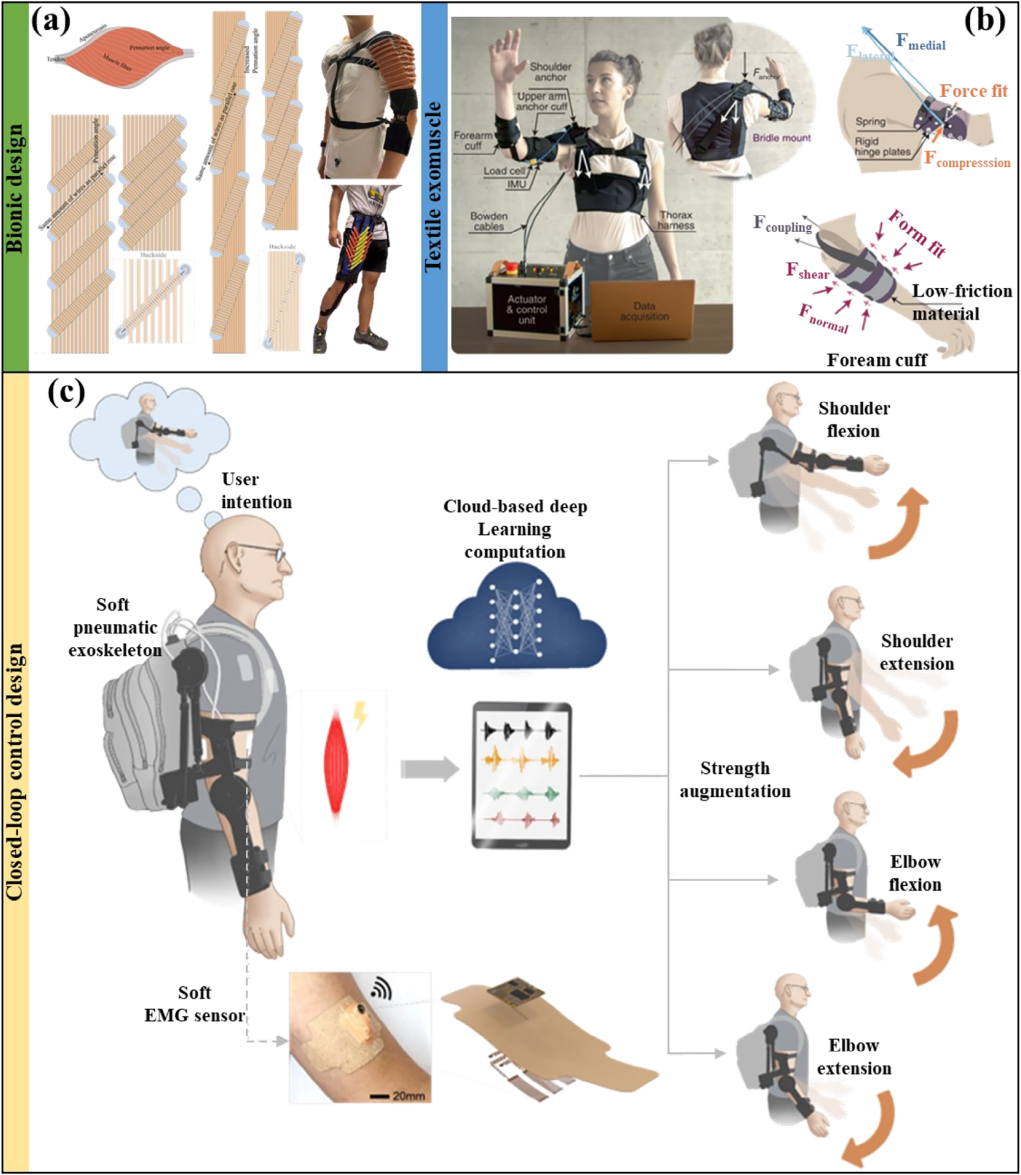
a) Fluid‐driven high‐performance artificial muscle that mimics skeletal muscle myometrium. Reproduced with permission.^[^ ^260^ ^]^ Copyright 2023, Wiley. b) Wearable textile exoskeleton to assist shoulder movement. Reproduced with permission.^[^ ^262^ ^]^ Copyright 2022, Springer Nature. c) Intent‐driven prediction of upper limb exoskeleton closed‐loop system architecture. Reproduced with permission.^[^ ^268^ ^]^ Copyright 2024, Springer Nature.

Muscle atrophy can reduce human strength. In addition to using ES or drug assistance, exoskeletons can also reduce the burden on the body and enhance human athletic ability and strength.^[^ ^258^, ^259^ ^]^ ExoMuscles (soft wearable actuators) used for muscle atrophy rehabilitation treatment are the exoskeletons we focus on. To improve the efficiency, wearability, and lightweight of such systems, bionic structure^[^ ^260^, ^261^ ^]^ (sliding filament hypothesis, muscle movement), flexible material^[^ ^262^, ^263^, ^264^, ^265^, ^266^ ^]^ (elastomers, fabrics) selection, and closed‐loop control strategies^[^ ^267^, ^268^ ^]^ are receiving increasing attention. ExoMuscles, designed with reference to the biological structure or movement mechanism of nature, are more in line with the physiological characteristics of the human body. They are also smoother and more efficient during operation. For example, inspired by skeletal muscle myometrium, Xie Disheng et al.^[^ ^260^ ^]^ designed a freely configurable high‐performance artificial muscle (**Figure** **10**a) with an actuation stress of 0.41–0.9 MPa.

In addition to providing mighty power, high‐quality ExoMuscles should also be easy to wear and comfortable. Unsurprisingly, textiles have become the ideal platform for carrying ExoMuscles due to their flexibility and breathability.^[^ ^262^, ^264^, ^269^ ^]^ Figure 10b shows a motor traction system using textiles as the operating platform of ExoMuscle.^[^ ^262^ ^]^ It successfully delayed the muscle fatigue time of patients with muscular dystrophy by 256.4 s (61.5%) and patients with spinal cord injury by 450.6 s (210.3%). However, the control unit is not portable and will affect the wearer's daily activities at home. Moreover, wearable devices use textiles as pneumatic actuators, restoring arm function in ALS patients.^[^ ^264^ ^]^ To meet complex movement tasks, precise motion control and dynamic adjustment of ExoMuscles are required, and closed‐loop control systems provide the possibility for breakthroughs. There are many ways to achieve closed‐loop perception, including EMG,^[^ ^268^ ^]^ IMU,^[^ ^261^, ^269^ ^]^ pressure sensors,^[^ ^262^, ^264^, ^267^ ^]^ etc. Lee, Jinwoo, et al.^[^ ^268^ ^]^ combined EMG with machine learning and pneumatic ExoMuscles (as shown in Figure 10c) to predict the movements of four upper limb joints at a response rate of 500–550 ms with an accuracy of 96.2% and reduced human muscle activity by 3.7 times. However, myoelectric signals disturbed by muscle fatigue or spasm may cause misjudgment of closed‐loop control. More information on the excellent performance of ExoMuscles for muscle atrophy assistance and rehabilitation is listed in **Table** **5**. This auxiliary treatment for muscle atrophy is limited to the early stages of atrophy and will make the patient's movements less natural.

**Table 5:** Summary of the characteristics of the extramuscular system for the assistance or rehabilitation of muscle atrophy.

Assistance/rehabilitation area	Materials	Weight	Power resources	Sensing unit	Refs.
Skeletal muscle	Polyurethane+thin filaments	–	Fluid	–	[260]
Upper‐extremity	Exosuit	–	Electric	IMU	[261]
Shoulder	Textile	0.52 kg (textile interface)	Electric	IMU+ load cell	[262]
Hand	Elastomer+textile	122 to 149 g (only glove)	Pneumatic	–	[263]
Shoulder	Textile		Pneumatic		[264]
Hand	Polyurethane+TiNi‐alloy	0.49 kg	Electric	Bending sensors	[267]
Upper‐limb	Carbon fiber+silicone+polyester+aluminum+	4.7 kg + 670 g	Pneumatic	EMG	[268]
Upper‐limb	Textile	–	Pneumatic	IMU	[269]

## 5 Electrode–Tissue Interfaces

The above review shows that electrodes are vital in monitoring and treating muscle atrophy. Although improving and developing algorithms can help process physiological signals, this also depends on acquiring high‐quality electrical signals. Transcutaneous, implantable, or drug delivery for muscle atrophy also depends on a high‐quality electrode–tissue interface. Otherwise, the therapeutic effect will be significantly reduced. Therefore, we will provide a general review of advanced electrode–tissue interface development methods. The treatment of interfaces involves complex bio‐mechanical‐electrical problems, and various techniques are currently used.

**Figure 11:**
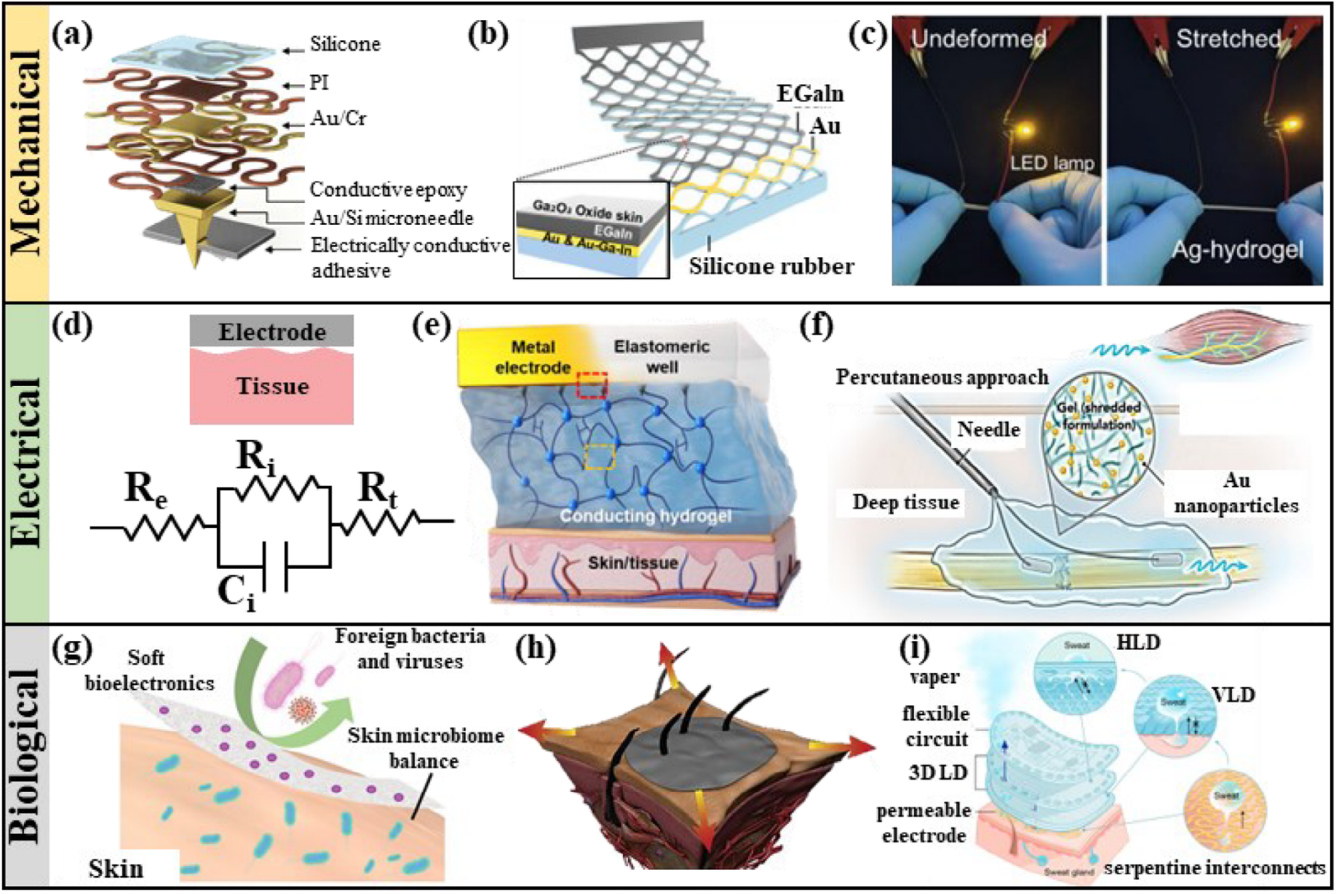
a) Stretchable microneedle adhesive patch for reliable electrophysiological sensing. Reproduced with permission.^[^ ^271^ ^]^ Copyright 2024, Science. b) Highly stretchable and strain‐insensitive liquid metal‐based elastic electrodes. Reproduced with permission.^[^ ^274^ ^]^ Copyright 2023, Wiley. c) Highly conductive hydrogel that is not affected by stretching lights up LEDs. Reproduced with permission.^[^ ^277^ ^]^ Copyright 2023, Wiley. d) Schematic diagram of the equivalent circuit at the electrode–tissue interface (*R*_t_–Tissue resistance, *R*_i_–Interface resistance, *R*_e_–Electrode resistance, *C*_i_–Capacitance at the interface). e) Highly conductive hydrogel that effectively reduces interfacial resistance. Reproduced with permission.^[^ ^279^ ^]^ Copyright 2024, Science. f) Rapidly cured in situ conductive electrotherapy scaffolds reduce interfacial resistance and enhance muscle regeneration. Reproduced with permission.^[^ ^282^ ^]^ Copyright 2023, Elsevier. g) Skin‐interfaced bioelectronics with biocompatibility and antimicrobial properties. Reproduced with permission.^[^ ^287^ ^]^ Copyright 2023, Science. h) Schematic diagram of viscoelastic dry electrodes that are not affected by hairy skin. Reproduced with permission.^[^ ^288^ ^]^ Copyright 2023, Wiley. i) Wearable device that enables stable ECG monitoring after sweating. Reproduced with permission.^[^ ^289^ ^]^ Copyright 2024, Springer Nature.

Designing a reliable and stable electrode–tissue interface is a massive challenge regarding mechanical properties. Specific structural design^[^ ^270^ ^]^ or electrode routing^[^ ^271^, ^272^ ^]^ (**Figure** **11**a,b) can protect the sensor from stress or strain interference, making the electrical signal reliable. In addition, selecting specific materials (such as brittle materials,^[^ ^273^ ^]^ liquid metal,^[^ ^274^ ^]^ etc.) can also avoid interference caused by deformation. The self‐powered sutures integrate the electrode fixation and ES system into one.^[^ ^275^ ^]^ Highly conductive stretch‐resistant hydrogels (Figure 11c) also provide a solution for developing electrode–tissue interfaces with stable mechanical properties.^[^ ^276^, ^277^, ^278^ ^]^

In addition to excellent mechanical properties, the electrical properties at the interface are also critical for acquiring high‐quality electrical signals and releasing current/voltage. The impedance at the electrode–tissue interface can be equivalent to the circuit shown in Figure 11d, which includes the electrode resistance, the resistance and capacitance at the interface, and the tissue resistance. From the schematic diagram, we can see only two ways to reduce interface impedance: 1) Use a medium with good conductivity to fill the gap at the interface^[^ ^276^, ^279^, ^280^ ^]^ (Figure 11e), 2) Develop adaptive electrodes to ensure complete contact between the electrode and the tissue^[^ ^281^, ^282^, ^283^, ^284^, ^285^ ^]^ (Figure 11f). The filling medium used in the former is mostly gel, but its characteristics are easily affected by changes in body fluids, and the physical sensation is poor. In situ phase change materials (the majority are liquid–solid) are mostly the latter choice. Although the phase change can depend on the stimulation of body fluids, currently, most of these electrodes rely on external stimulation such as temperature, light, or water.

When electrodes are placed in tissues, in addition to satisfying the above two characteristics, they must also have specific physiological characteristics depending on the placement location.^[^ ^286^ ^]^ Electrodes in contact with the human body need to be biocompatible. In addition to passively selecting materials with this property, materials with antibacterial properties can also be mixed into the electrodes^[^ ^287^ ^]^ (Figure 11g) to achieve active defense functions. Electrodes placed on the skin's surface may also be affected by hair growth or sweat, making long‐term stable monitoring or treatment challenging. Tian Qiong et al.^[^ ^288^ ^]^ designed a skin‐adaptive viscoelastic dry electrode, as shown in Figure 11h, and monitored the ECG for up to 60 days. Zhang Binbin et al.^[^ ^289^ ^]^ designed a three‐dimensional liquid diode (Figure 11i) with a maximum flow rate of 11.6 mL cm^−2^ min^−1^ to avoid the influence of sweat on ECG signals.

## 6 Summary and Outlook

We first summarized the diseases associated with muscle atrophy. This disease will reduce muscle strength, volume, and mass, making it difficult to maintain daily life. However, our current knowledge of its pathways is limited. This greatly limits our understanding of the pathogenesis and makes it difficult to conduct accurate and effective prevention and treatment. Therefore, easy‐to‐wear home detection and treatment devices are necessary to diagnose and treat current muscle atrophy.

**Figure 12:**
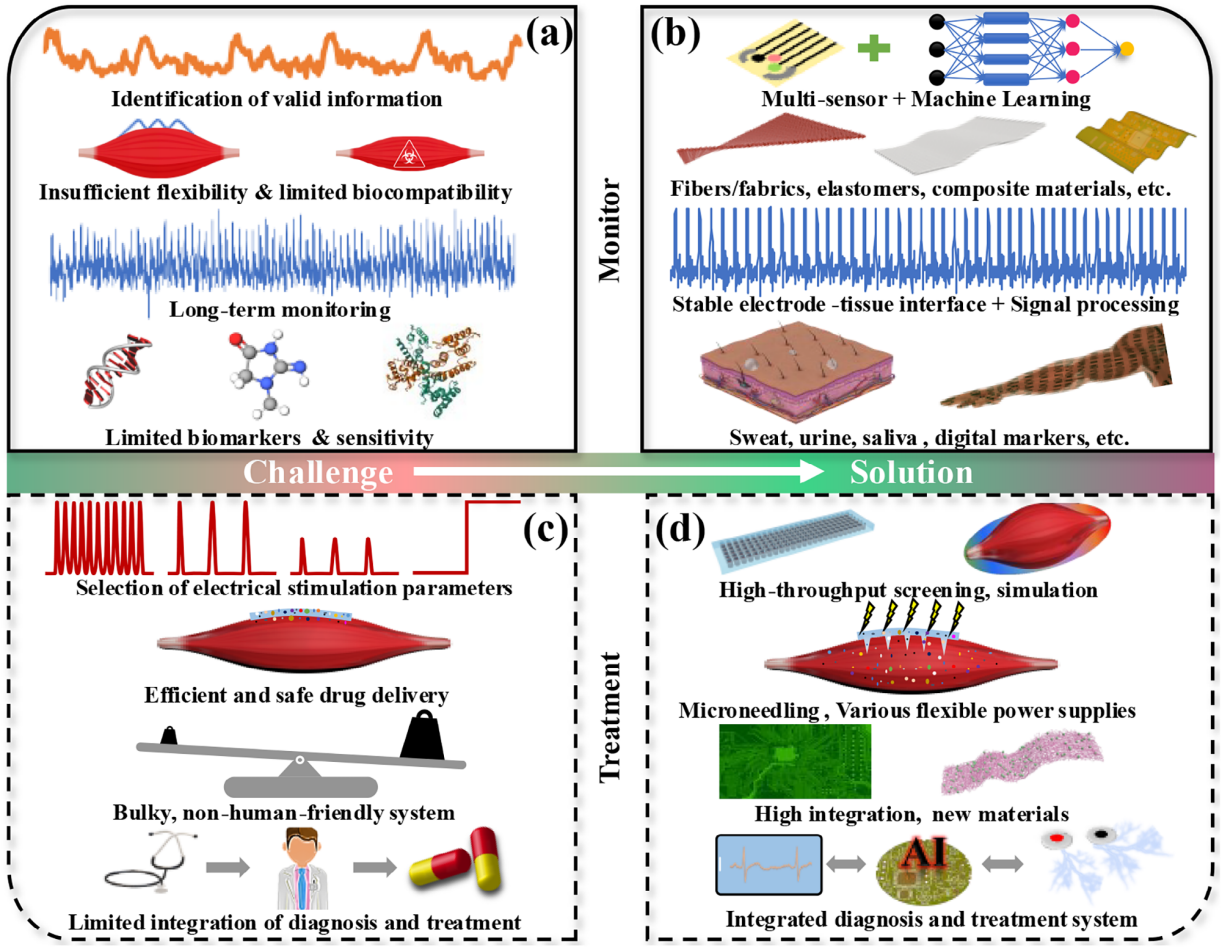
a,c) Challenges and b,d) potential solutions for the development of bioelectronics for home care of muscle atrophy.

We divide the current detection methods into four categories according to their working mechanism and characteristics: mechanical sensors, EMG, bioelectrical impedance, ultrasonic patches, and electrochemical sensors. All sensor systems suffer from limited information (precise quantification of muscle strength, size, mass, and composition) and difficulty maintaining long‐term stability. This makes it difficult to determine the cause of muscle atrophy. The flexibility of the electrodes of various sensors is also limited, which limits the application scenarios of wearable sensors and signal distortion. In addition, limited biomarkers and safe and efficient methods of extracting body fluids restrict the application of electrochemical sensors. To address the challenges mentioned above (**Figure** **12**a), we have proposed practical solutions (Figure 12b).

1. Integration of multi‐sensing technologies: Future devices should incorporate multiple sensing modalities,^[^ ^290^ ^]^ such as mechanical sensors, EMG, bioelectrical impedance, and electrochemical sensors, into a single platform. This integration would allow for comprehensive monitoring of muscle health, providing a more holistic view of muscle function and atrophy progression.
2. Biocompatible and flexible materials: Research should focus on developing new biocompatible and flexible materials, allowing for comfortable long‐term wear. Materials such as conductive polymers (such as PDA, PPP, PEO, PEDOT, etc.) or advanced textiles (such as Ag‐coated yarn, Silk fibroin, PVDF) that mimic the mechanical properties of human skin and muscle can improve user compliance and data accuracy.
3. Acquisition of high‐quality biological signals: Implementing advanced machine learning algorithms can enhance the data analysis capabilities of sensing technologies. These algorithms should be designed to identify patterns and correlations in the data that may indicate early signs of muscle atrophy so that timely intervention measures can be taken. In addition, a stable electrode–tissue interface is the basis for long‐term stable signal acquisition.
4. Enhanced biomarker detection: Efforts should be made to identify and validate new biomarkers for muscle atrophy that can be easily detected through non‐invasive methods (such as sweat, saliva, urine, or digital biomarkers).

Until now, there is no practical way to cure muscle atrophy, so the prevention and treatment of muscle atrophy using wearable bioelectronics is full of challenges (Figure 12c). Although electrotherapy's effectiveness in treating muscle atrophy has been confirmed, there is currently no consensus on the electrotherapy parameters (frequency, amplitude, waveform, etc.) to be used. This is mainly due to individual differences and the non‐standardization of electrotherapy. To achieve uniformity, continuous experimental exploration is needed. For the electronic drug delivery methods mentioned in the paper, high‐voltage electroporation may cause irreversible damage to cells, while low‐current iontophoresis has a limited reach depth. In addition, the lightweight and intelligent wearable systems need to be improved. Developing an intelligent integrated diagnosis and treatment system is necessary to reduce the professional requirements for home care of muscle atrophy. Accordingly, we also propose potential solutions for home treatment of muscle atrophy (Figure 12d).

1. High‐throughput or rapid screening platforms: Establish a platform for rapid screening of electrotherapy parameters^[^ ^291^ ^]^ or drug types in the laboratory (patient cells or digital muscle parameters), and promptly provide feedback to patients at home for setting. This high‐throughput or simulation platform that does not require patients to be present can significantly save time and achieve efficient treatment.
2. Innovative and personalized treatment system: Future technologies should explore novel drug delivery methods, such as microneedles or smart patches that can release medications in a controlled manner. These systems should be designed to minimize discomfort and maximize the therapeutic effect, targeting muscle tissue directly.
3. Lightweight, wearable therapeutic devices: Develop portable therapeutic devices that can be easily used at home. These devices should allow patients to customize treatment parameters (frequency, intensity, duration) based on individual needs and responses, with guidance from a healthcare provider.
4. Integration of intelligent remote diagnosis and treatment system: Establish a data sharing platform to enable artificial intelligence algorithms to learn monitoring data, diagnosis, and treatment plans. Combined with the judgment of professional doctors, an integrated intelligent system for medical data collection and treatment plan formulation will be realized.
5. ES interface materials: Metals (Au, Ag/AgCl), conductive polymers, semiconductors (MnO_2_, TiN, MoS_2_), and composites (conductive hydrogel, carbon‐based materials) have been widely used in ES delivery. These materials have unique advantages, so ES should be selected according to the specific requirements of the intended application. In principle, an ideal ES system should have excellent electrical properties, strict mechanical properties, good tissue adhesion, unique bioactivity (e.g., biocompatibility and antibacterial efficacy), biodegradability, and some other key properties (e.g., appropriate permeability, swelling, and self‐healing ability).

In addition to the above‐mentioned technical difficulties, there are many challenges in applying laboratory products to patients' homes.

1. Calibration and maintenance: Equipment often requires regular calibration and maintenance to ensure accuracy and reliability. Patients may lack the resources or knowledge to perform these tasks, leading to potential inaccuracies in monitoring data.
2. Regulatory and compliance issues: Home‐use medical devices must comply with regulatory standards, which can be challenging for laboratory technologies not initially designed for this purpose. Navigating the regulatory landscape can delay the development and approval of home monitoring solutions.
3. User compliance and engagement: Encouraging patients to use equipment at home consistently can be challenging. Factors such as user fatigue, lack of motivation, or difficulty understanding technology use can lead to inconsistent monitoring and treatment adherence.
4. Patient education and support: Effective use of appliances at home requires adequate patient education and ongoing support. Developing comprehensive educational resources and support systems can be resource‐intensive and may not always be feasible.

To solve the above problems, manufacturers need to reduce the complexity of instrument operation and increase their reliability; patients need to receive professional training to reduce operating errors and achieve safe operation; the government needs to establish a regulatory system to avoid data leakage; and medical staff need to strengthen professional guidance to avoid safety accidents. In short, the efficient use of instruments and resources for home monitoring and treatment of muscle atrophy requires the participation and efforts of multiple parties.

## Conflict of Interest

The authors declare no conflict of interest.
